# Supplementary material for: Interaction between Microalgae P. tricornutum and Bacteria Thalassospira sp. for Removal of Bisphenols from Conditioned Media
Source: Int J Mol Sci. 2022 Jul 30;23(15):8447. doi: 10.3390/ijms23158447 (PMC9369128; doi:10.3390/ijms23158447)
Supplement: Supplementary file 1 [file ijms-23-08447-s001.zip › ijms-1796904-supplementary.pdf]

International Journal of
Molecular Sciences

MDPI

Supplementary Material Škufca et al.

Table S1. Bisphenol compound abbreviations, International Union of Pure and Applied Chemistry (IUPAC) names and Chemical Abstract Services (CAS) identifiers.

Abbreviation	IUPAC name	CAS
BPA	4-[2-(4-hydroxyphenyl)propan-2-yl]phenol	80-05-7
2,2′-BPF	2-[(2-hydroxyphenyl)methyl]phenol	2467-02-9
2,4′-BPF	2-[(4-hydroxyphenyl)methyl]phenol	2467-03-0
4,4′-BPF	4-[(4-hydroxyphenyl)methyl]phenol	620-92-8
BP26DM	4-[2-(4-hydroxy-3,5-dimethylphenyl)propan-2-yl]-2,6-dimethylphenol	5613-46-7
BPAF	4-[1,1,1,3,3,3-Hexafluoro-2-(4-hydroxyphenyl)propan-2-yl]phenol	1478-61-1
BPAP	4-[1-(4-hydroxyphenyl)-1-phenylethyl]phenol	1571-75-1
BPB	4-[2-(4-hydroxyphenyl)butan-2-yl]phenol	77-40-7
BPBP	4-[(4-hydroxyphenyl)-diphenylmethyl]phenol	1844-01-5
BPC	4-[2-(4-hydroxy-3-methylphenyl)propan-2-yl]-2-methylphenol	79-97-0
BPC2	4-[2,2-dichloro-1-(4-hydroxyphenyl)ethenyl]phenol	14868-03-2
BPE	4-[1-(4-hydroxyphenyl)ethyl]phenol	2081-08-5
BPFL	4-[9-(4-hydroxyphenyl)fluoren-9-yl]phenol	3236-71-3
BPM	4-[2-[3-[2-(4-hydroxyphenyl)propan-2-yl]phenyl]propan-2-yl]phenol	13595-25-0
BPP	4-[2-[4-[2-(4-hydroxyphenyl)propan-2-yl]phenyl]propan-2-yl]phenol	2167-51-3
BPPH	4-[2-(4-hydroxy-3-phenylphenyl)propan-2-yl]-2-phenylphenol	24038-68-4
BPS	4-(4-hydroxyphenyl)sulfonylphenol	80-09-1
BPZ	4-[1-(4-hydroxyphenyl)cyclohexyl]phenol	843-55-0

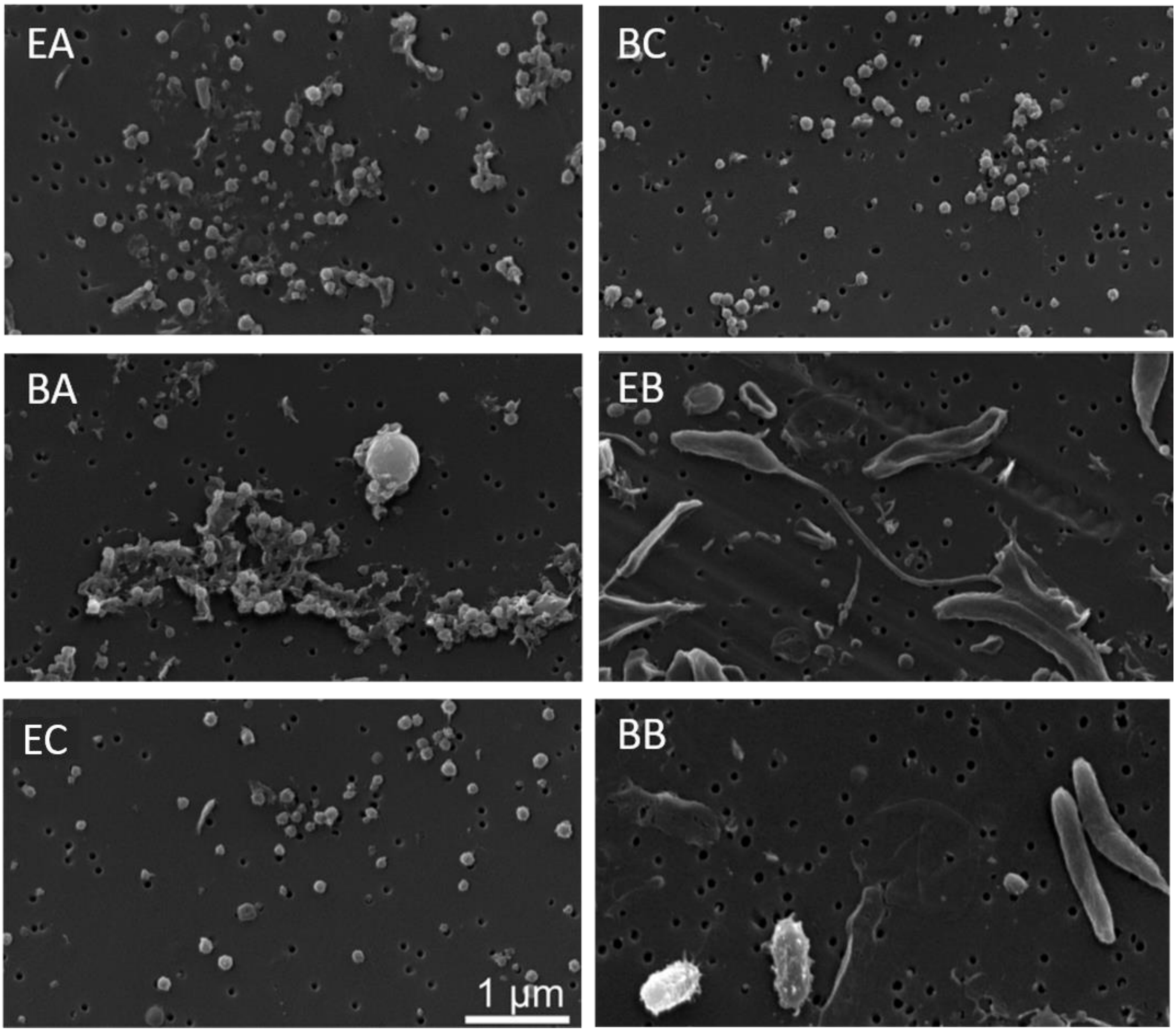

Figure S1. **A**dditional SEM images of SCP isolates from EA, BA, EC, BC, EB and BB cultures. Experimental series (addition of BPs): axenic microalgae (EA), co-culture of microalgae and bacteria (EC), and bacterial culture (EB). Blank control series (no addition of BPs): axenic microalgae (BA), co-culture of microalgae and bacteria (BC), and bacterial culture (BB).

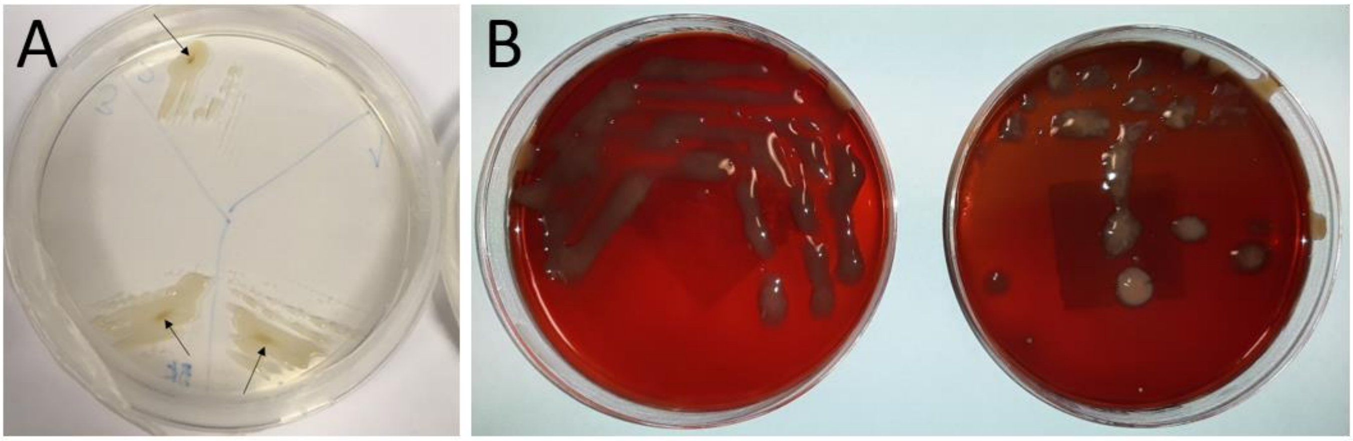

Figure S2. A: Co-culture sample grown on an 1% MW-LB agar, showing growth of bacteria, with microalgae *P. tricornutum* growth in the middle of bacterial growth (arrows). **B:** pre-cultures of bacteria on BD Columbia agar containing 5% sheep blood.

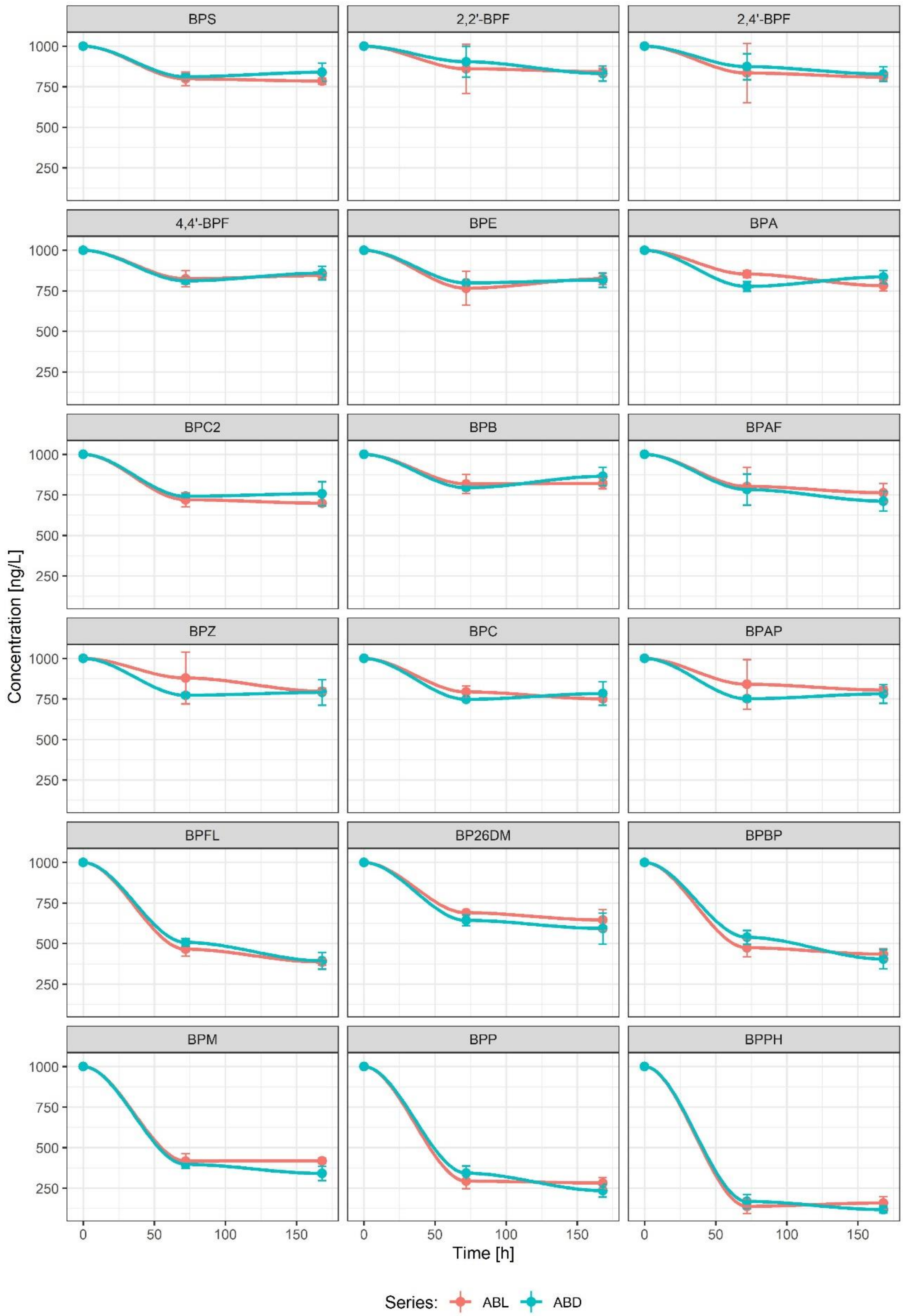

Figure S3. Concentration of 18 BPs in light (ABL) and dark (ABD) abiotic conditions. The mean (dot) and standard deviation (error-bars) are shown (n=3). Compounds are arranged from lowest (BPS, log P = 2.3) to highest (BPPH, log P = 7.3) log P value.

Table S2. Raw data on bisphenol removal

Compound	Sample	Time in days	Time in h	Reactor	Type	Aqueous phase	Biomass	Removal (aq.)	Total removal	Difference in removal	Algae removal	Small	Ratio
22BPF	t0_ABS1	0	0	1	ABS	1000		0,0%	0,0%	0,0%	0,00008	0,16551	0,000483
BPAF	t0_ABS1	0	0	1	ABS	1000		0,0%	0,0%	0,0%	0,00008	0,16551	0,000483
24BPF	t0_ABS1	0	0	1	ABS	1000		0,0%	0,0%	0,0%	0,00008	0,16551	0,000483
44BPF	t0_ABS1	0	0	1	ABS	1000		0,0%	0,0%	0,0%	0,00008	0,16551	0,000483
BPE	t0_ABS1	0	0	1	ABS	1000		0,0%	0,0%	0,0%	0,00008	0,16551	0,000483
BPA	t0_ABS1	0	0	1	ABS	1000		0,0%	0,0%	0,0%	0,00008	0,16551	0,000483
BPC	t0_ABS1	0	0	1	ABS	1000		0,0%	0,0%	0,0%	0,00008	0,16551	0,000483
BPB	t0_ABS1	0	0	1	ABS	1000		0,0%	0,0%	0,0%	0,00008	0,16551	0,000483
BP26DM	t0_ABS1	0	0	1	ABS	1000		0,0%	0,0%	0,0%	0,00008	0,16551	0,000483
BPC2	t0_ABS1	0	0	1	ABS	1000		0,0%	0,0%	0,0%	0,00008	0,16551	0,000483
BPZ	t0_ABS1	0	0	1	ABS	1000		0,0%	0,0%	0,0%	0,00008	0,16551	0,000483
BPS	t0_ABS1	0	0	1	ABS	1000		0,0%	0,0%	0,0%	0,00008	0,16551	0,000483
BPAP	t0_ABS1	0	0	1	ABS	1000		0,0%	0,0%	0,0%	0,00008	0,16551	0,000483
BPM	t0_ABS1	0	0	1	ABS	1000		0,0%	0,0%	0,0%	0,00008	0,16551	0,000483
BPP	t0_ABS1	0	0	1	ABS	1000		0,0%	0,0%	0,0%	0,00008	0,16551	0,000483
BPBP	t0_ABS1	0	0	1	ABS	1000		0,0%	0,0%	0,0%	0,00008	0,16551	0,000483
BPPH	t0_ABS1	0	0	1	ABS	1000		0,0%	0,0%	0,0%	0,00008	0,16551	0,000483
BPFL	t0_ABS1	0	0	1	ABS	1000		0,0%	0,0%	0,0%	0,00008	0,16551	0,000483
22BPF	t0_ABS2	0	0	2	ABS	1000		0,0%	0,0%	0,0%	0,0002	0,17188	0,001164
BPAF	t0_ABS2	0	0	2	ABS	1000		0,0%	0,0%	0,0%	0,0002	0,17188	0,001164
24BPF	t0_ABS2	0	0	2	ABS	1000		0,0%	0,0%	0,0%	0,0002	0,17188	0,001164
44BPF	t0_ABS2	0	0	2	ABS	1000		0,0%	0,0%	0,0%	0,0002	0,17188	0,001164
BPE	t0_ABS2	0	0	2	ABS	1000		0,0%	0,0%	0,0%	0,0002	0,17188	0,001164
BPA	t0_ABS2	0	0	2	ABS	1000		0,0%	0,0%	0,0%	0,0002	0,17188	0,001164
BPC	t0_ABS2	0	0	2	ABS	1000		0,0%	0,0%	0,0%	0,0002	0,17188	0,001164
BPB	t0_ABS2	0	0	2	ABS	1000		0,0%	0,0%	0,0%	0,0002	0,17188	0,001164
BP26DM	t0_ABS2	0	0	2	ABS	1000		0,0%	0,0%	0,0%	0,0002	0,17188	0,001164
BPC2	t0_ABS2	0	0	2	ABS	1000		0,0%	0,0%	0,0%	0,0002	0,17188	0,001164
BPZ	t0_ABS2	0	0	2	ABS	1000		0,0%	0,0%	0,0%	0,0002	0,17188	0,001164
BPS	t0_ABS2	0	0	2	ABS	1000		0,0%	0,0%	0,0%	0,0002	0,17188	0,001164
BPAP	t0_ABS2	0	0	2	ABS	1000		0,0%	0,0%	0,0%	0,0002	0,17188	0,001164
BPM	t0_ABS2	0	0	2	ABS	1000		0,0%	0,0%	0,0%	0,0002	0,17188	0,001164
BPP	t0_ABS2	0	0	2	ABS	1000		0,0%	0,0%	0,0%	0,0002	0,17188	0,001164
BPBP	t0_ABS2	0	0	2	ABS	1000		0,0%	0,0%	0,0%	0,0002	0,17188	0,001164
BPPH	t0_ABS2	0	0	2	ABS	1000		0,0%	0,0%	0,0%	0,0002	0,17188	0,001164
BPFL	t0_ABS2	0	0	2	ABS	1000		0,0%	0,0%	0,0%	0,0002	0,17188	0,001164
22BPF	t0_ABS3	0	0	3	ABS	1000		0,0%	0,0%	0,0%	0,00012	0,12879	0,000932
BPAF	t0_ABS3	0	0	3	ABS	1000		0,0%	0,0%	0,0%	0,00012	0,12879	0,000932
24BPF	t0_ABS3	0	0	3	ABS	1000		0,0%	0,0%	0,0%	0,00012	0,12879	0,000932
44BPF	t0_ABS3	0	0	3	ABS	1000		0,0%	0,0%	0,0%	0,00012	0,12879	0,000932
BPE	t0_ABS3	0	0	3	ABS	1000		0,0%	0,0%	0,0%	0,00012	0,12879	0,000932
BPA	t0_ABS3	0	0	3	ABS	1000		0,0%	0,0%	0,0%	0,00012	0,12879	0,000932
BPC	t0_ABS3	0	0	3	ABS	1000		0,0%	0,0%	0,0%	0,00012	0,12879	0,000932
BPB	t0_ABS3	0	0	3	ABS	1000		0,0%	0,0%	0,0%	0,00012	0,12879	0,000932
BP26DM	t0_ABS3	0	0	3	ABS	1000		0,0%	0,0%	0,0%	0,00012	0,12879	0,000932
BPC2	t0_ABS3	0	0	3	ABS	1000		0,0%	0,0%	0,0%	0,00012	0,12879	0,000932
BPZ	t0_ABS3	0	0	3	ABS	1000		0,0%	0,0%	0,0%	0,00012	0,12879	0,000932
BPS	t0_ABS3	0	0	3	ABS	1000		0,0%	0,0%	0,0%	0,00012	0,12879	0,000932
BPAP	t0_ABS3	0	0	3	ABS	1000		0,0%	0,0%	0,0%	0,00012	0,12879	0,000932
BPM	t0_ABS3	0	0	3	ABS	1000		0,0%	0,0%	0,0%	0,00012	0,12879	0,000932
BPP	t0_ABS3	0	0	3	ABS	1000		0,0%	0,0%	0,0%	0,00012	0,12879	0,000932
BPBP	t0_ABS3	0	0	3	ABS	1000		0,0%	0,0%	0,0%	0,00012	0,12879	0,000932
BPPH	t0_ABS3	0	0	3	ABS	1000		0,0%	0,0%	0,0%	0,00012	0,12879	0,000932
BPFL	t0_ABS3	0	0	3	ABS	1000		0,0%	0,0%	0,0%	0,00012	0,12879	0,000932
22BPF	t0_ABT1	0	0	1	ABT	1000		0,0%	0,0%	0,0%	0,00004	0,05124	0,000781
BPAF	t0_ABT1	0	0	1	ABT	1000		0,0%	0,0%	0,0%	0,00004	0,05124	0,000781
24BPF	t0_ABT1	0	0	1	ABT	1000		0,0%	0,0%	0,0%	0,00004	0,05124	0,000781
44BPF	t0_ABT1	0	0	1	ABT	1000		0,0%	0,0%	0,0%	0,00004	0,05124	0,000781
BPE	t0_ABT1	0	0	1	ABT	1000		0,0%	0,0%	0,0%	0,00004	0,05124	0,000781
BPA	t0_ABT1	0	0	1	ABT	1000		0,0%	0,0%	0,0%	0,00004	0,05124	0,000781
BPC	t0_ABT1	0	0	1	ABT	1000		0,0%	0,0%	0,0%	0,00004	0,05124	0,000781
BPB	t0_ABT1	0	0	1	ABT	1000		0,0%	0,0%	0,0%	0,00004	0,05124	0,000781
BP26DM	t0_ABT1	0	0	1	ABT	1000		0,0%	0,0%	0,0%	0,00004	0,05124	0,000781
BPC2	t0_ABT1	0	0	1	ABT	1000		0,0%	0,0%	0,0%	0,00004	0,05124	0,000781
BPZ	t0_ABT1	0	0	1	ABT	1000		0,0%	0,0%	0,0%	0,00004	0,05124	0,000781
BPS	t0_ABT1	0	0	1	ABT	1000		0,0%	0,0%	0,0%	0,00004	0,05124	0,000781
BPAP	t0_ABT1	0	0	1	ABT	1000		0,0%	0,0%	0,0%	0,00004	0,05124	0,000781
BPM	t0_ABT1	0	0	1	ABT	1000		0,0%	0,0%	0,0%	0,00004	0,05124	0,000781
BPP	t0_ABT1	0	0	1	ABT	1000		0,0%	0,0%	0,0%	0,00004	0,05124	0,000781
BPBP	t0_ABT1	0	0	1	ABT	1000		0,0%	0,0%	0,0%	0,00004	0,05124	0,000781
BPPH	t0_ABT1	0	0	1	ABT	1000		0,0%	0,0%	0,0%	0,00004	0,05124	0,000781
BPFL	t0_ABT1	0	0	1	ABT	1000		0,0%	0,0%	0,0%	0,00004	0,05124	0,000781
22BPF	t0_ABT2	0	0	2	ABT	1000		0,0%	0,0%	0,0%	0,00016	0,10923	0,001465
BPAF	t0_ABT2	0	0	2	ABT	1000		0,0%	0,0%	0,0%	0,00016	0,10923	0,001465
24BPF	t0_ABT2	0	0	2	ABT	1000		0,0%	0,0%	0,0%	0,00016	0,10923	0,001465
44BPF	t0_ABT2	0	0	2	ABT	1000		0,0%	0,0%	0,0%	0,00016	0,10923	0,001465
BPE	t0_ABT2	0	0	2	ABT	1000		0,0%	0,0%	0,0%	0,00016	0,10923	0,001465
BPA	t0_ABT2	0	0	2	ABT	1000		0,0%	0,0%	0,0%	0,00016	0,10923	0,001465
BPC	t0_ABT2	0	0	2	ABT	1000		0,0%	0,0%	0,0%	0,00016	0,10923	0,001465
BPB	t0_ABT2	0	0	2	ABT	1000		0,0%	0,0%	0,0%	0,00016	0,10923	0,001465
BP26DM	t0_ABT2	0	0	2	ABT	1000		0,0%	0,0%	0,0%	0,00016	0,10923	0,001465
BPC2	t0_ABT2	0	0	2	ABT	1000		0,0%	0,0%	0,0%	0,00016	0,10923	0,001465
BPZ	t0_ABT2	0	0	2	ABT	1000		0,0%	0,0%	0,0%	0,00016	0,10923	0,001465
BPS	t0_ABT2	0	0	2	ABT	1000		0,0%	0,0%	0,0%	0,00016	0,10923	0,001465

BPAP	t0_ABT2	0	0	2	ABT	1000	0,0%	0,0%	0,0%	0,00016	0,10923	0,001465
BPM	t0_ABT2	0	0	2	ABT	1000	0,0%	0,0%	0,0%	0,00016	0,10923	0,001465
BPP	t0_ABT2	0	0	2	ABT	1000	0,0%	0,0%	0,0%	0,00016	0,10923	0,001465
BPBP	t0_ABT2	0	0	2	ABT	1000	0,0%	0,0%	0,0%	0,00016	0,10923	0,001465
BPPH	t0_ABT2	0	0	2	ABT	1000	0,0%	0,0%	0,0%	0,00016	0,10923	0,001465
BPFL	t0_ABT2	0	0	2	ABT	1000	0,0%	0,0%	0,0%	0,00016	0,10923	0,001465
22BPF	t0_ABT3	0	0	3	ABT	1000	0,0%	0,0%	0,0%	0,00036	0,12933	0,002784
BPAF	t0_ABT3	0	0	3	ABT	1000	0,0%	0,0%	0,0%	0,00036	0,12933	0,002784
24BPF	t0_ABT3	0	0	3	ABT	1000	0,0%	0,0%	0,0%	0,00036	0,12933	0,002784
44BPF	t0_ABT3	0	0	3	ABT	1000	0,0%	0,0%	0,0%	0,00036	0,12933	0,002784
BPE	t0_ABT3	0	0	3	ABT	1000	0,0%	0,0%	0,0%	0,00036	0,12933	0,002784
BPA	t0_ABT3	0	0	3	ABT	1000	0,0%	0,0%	0,0%	0,00036	0,12933	0,002784
BPC	t0_ABT3	0	0	3	ABT	1000	0,0%	0,0%	0,0%	0,00036	0,12933	0,002784
BPB	t0_ABT3	0	0	3	ABT	1000	0,0%	0,0%	0,0%	0,00036	0,12933	0,002784
BP26DM	t0_ABT3	0	0	3	ABT	1000	0,0%	0,0%	0,0%	0,00036	0,12933	0,002784
BPC2	t0_ABT3	0	0	3	ABT	1000	0,0%	0,0%	0,0%	0,00036	0,12933	0,002784
BPZ	t0_ABT3	0	0	3	ABT	1000	0,0%	0,0%	0,0%	0,00036	0,12933	0,002784
BPS	t0_ABT3	0	0	3	ABT	1000	0,0%	0,0%	0,0%	0,00036	0,12933	0,002784
BPAP	t0_ABT3	0	0	3	ABT	1000	0,0%	0,0%	0,0%	0,00036	0,12933	0,002784
BPM	t0_ABT3	0	0	3	ABT	1000	0,0%	0,0%	0,0%	0,00036	0,12933	0,002784
BPP	t0_ABT3	0	0	3	ABT	1000	0,0%	0,0%	0,0%	0,00036	0,12933	0,002784
BPBP	t0_ABT3	0	0	3	ABT	1000	0,0%	0,0%	0,0%	0,00036	0,12933	0,002784
BPPH	t0_ABT3	0	0	3	ABT	1000	0,0%	0,0%	0,0%	0,00036	0,12933	0,002784
BPFL	t0_ABT3	0	0	3	ABT	1000	0,0%	0,0%	0,0%	0,00036	0,12933	0,002784
22BPF	t0_BA1	0	0	1	BA	1000	0,0%	0,0%	0,0%	1,03335	0,24428	4,230187
BPAF	t0_BA1	0	0	1	BA	1000	0,0%	0,0%	0,0%	1,03335	0,24428	4,230187
24BPF	t0_BA1	0	0	1	BA	1000	0,0%	0,0%	0,0%	1,03335	0,24428	4,230187
44BPF	t0_BA1	0	0	1	BA	1000	0,0%	0,0%	0,0%	1,03335	0,24428	4,230187
BPE	t0_BA1	0	0	1	BA	1000	0,0%	0,0%	0,0%	1,03335	0,24428	4,230187
BPA	t0_BA1	0	0	1	BA	1000	0,0%	0,0%	0,0%	1,03335	0,24428	4,230187
BPC	t0_BA1	0	0	1	BA	1000	0,0%	0,0%	0,0%	1,03335	0,24428	4,230187
BPB	t0_BA1	0	0	1	BA	1000	0,0%	0,0%	0,0%	1,03335	0,24428	4,230187
BP26DM	t0_BA1	0	0	1	BA	1000	0,0%	0,0%	0,0%	1,03335	0,24428	4,230187
BPC2	t0_BA1	0	0	1	BA	1000	0,0%	0,0%	0,0%	1,03335	0,24428	4,230187
BPZ	t0_BA1	0	0	1	BA	1000	0,0%	0,0%	0,0%	1,03335	0,24428	4,230187
BPS	t0_BA1	0	0	1	BA	1000	0,0%	0,0%	0,0%	1,03335	0,24428	4,230187
BPAP	t0_BA1	0	0	1	BA	1000	0,0%	0,0%	0,0%	1,03335	0,24428	4,230187
BPM	t0_BA1	0	0	1	BA	1000	0,0%	0,0%	0,0%	1,03335	0,24428	4,230187
BPP	t0_BA1	0	0	1	BA	1000	0,0%	0,0%	0,0%	1,03335	0,24428	4,230187
BPBP	t0_BA1	0	0	1	BA	1000	0,0%	0,0%	0,0%	1,03335	0,24428	4,230187
BPPH	t0_BA1	0	0	1	BA	1000	0,0%	0,0%	0,0%	1,03335	0,24428	4,230187
BPFL	t0_BA1	0	0	1	BA	1000	0,0%	0,0%	0,0%	1,03335	0,24428	4,230187
22BPF	t0_BA2	0	0	2	BA	1000	0,0%	0,0%	0,0%	1,03418	0,22503	4,595743
BPAF	t0_BA2	0	0	2	BA	1000	0,0%	0,0%	0,0%	1,03418	0,22503	4,595743
24BPF	t0_BA2	0	0	2	BA	1000	0,0%	0,0%	0,0%	1,03418	0,22503	4,595743
44BPF	t0_BA2	0	0	2	BA	1000	0,0%	0,0%	0,0%	1,03418	0,22503	4,595743
BPE	t0_BA2	0	0	2	BA	1000	0,0%	0,0%	0,0%	1,03418	0,22503	4,595743
BPA	t0_BA2	0	0	2	BA	1000	0,0%	0,0%	0,0%	1,03418	0,22503	4,595743
BPC	t0_BA2	0	0	2	BA	1000	0,0%	0,0%	0,0%	1,03418	0,22503	4,595743
BPB	t0_BA2	0	0	2	BA	1000	0,0%	0,0%	0,0%	1,03418	0,22503	4,595743
BP26DM	t0_BA2	0	0	2	BA	1000	0,0%	0,0%	0,0%	1,03418	0,22503	4,595743
BPC2	t0_BA2	0	0	2	BA	1000	0,0%	0,0%	0,0%	1,03418	0,22503	4,595743
BPZ	t0_BA2	0	0	2	BA	1000	0,0%	0,0%	0,0%	1,03418	0,22503	4,595743
BPS	t0_BA2	0	0	2	BA	1000	0,0%	0,0%	0,0%	1,03418	0,22503	4,595743
BPAP	t0_BA2	0	0	2	BA	1000	0,0%	0,0%	0,0%	1,03418	0,22503	4,595743
BPM	t0_BA2	0	0	2	BA	1000	0,0%	0,0%	0,0%	1,03418	0,22503	4,595743
BPP	t0_BA2	0	0	2	BA	1000	0,0%	0,0%	0,0%	1,03418	0,22503	4,595743
BPBP	t0_BA2	0	0	2	BA	1000	0,0%	0,0%	0,0%	1,03418	0,22503	4,595743
BPPH	t0_BA2	0	0	2	BA	1000	0,0%	0,0%	0,0%	1,03418	0,22503	4,595743
BPFL	t0_BA2	0	0	2	BA	1000	0,0%	0,0%	0,0%	1,03418	0,22503	4,595743
22BPF	t0_BA3	0	0	3	BA	1000	0,0%	0,0%	0,0%	1,0353	0,20894	4,955011
BPAF	t0_BA3	0	0	3	BA	1000	0,0%	0,0%	0,0%	1,0353	0,20894	4,955011
24BPF	t0_BA3	0	0	3	BA	1000	0,0%	0,0%	0,0%	1,0353	0,20894	4,955011
44BPF	t0_BA3	0	0	3	BA	1000	0,0%	0,0%	0,0%	1,0353	0,20894	4,955011
BPE	t0_BA3	0	0	3	BA	1000	0,0%	0,0%	0,0%	1,0353	0,20894	4,955011
BPA	t0_BA3	0	0	3	BA	1000	0,0%	0,0%	0,0%	1,0353	0,20894	4,955011
BPC	t0_BA3	0	0	3	BA	1000	0,0%	0,0%	0,0%	1,0353	0,20894	4,955011
BPB	t0_BA3	0	0	3	BA	1000	0,0%	0,0%	0,0%	1,0353	0,20894	4,955011
BP26DM	t0_BA3	0	0	3	BA	1000	0,0%	0,0%	0,0%	1,0353	0,20894	4,955011
BPC2	t0_BA3	0	0	3	BA	1000	0,0%	0,0%	0,0%	1,0353	0,20894	4,955011
BPZ	t0_BA3	0	0	3	BA	1000	0,0%	0,0%	0,0%	1,0353	0,20894	4,955011
BPS	t0_BA3	0	0	3	BA	1000	0,0%	0,0%	0,0%	1,0353	0,20894	4,955011
BPAP	t0_BA3	0	0	3	BA	1000	0,0%	0,0%	0,0%	1,0353	0,20894	4,955011
BPM	t0_BA3	0	0	3	BA	1000	0,0%	0,0%	0,0%	1,0353	0,20894	4,955011
BPP	t0_BA3	0	0	3	BA	1000	0,0%	0,0%	0,0%	1,0353	0,20894	4,955011
BPBP	t0_BA3	0	0	3	BA	1000	0,0%	0,0%	0,0%	1,0353	0,20894	4,955011
BPPH	t0_BA3	0	0	3	BA	1000	0,0%	0,0%	0,0%	1,0353	0,20894	4,955011
BPFL	t0_BA3	0	0	3	BA	1000	0,0%	0,0%	0,0%	1,0353	0,20894	4,955011
22BPF	t0_BB1	0	0	1	BB	1000	0,0%	0,0%	0,0%	0	0,43612	0
BPAF	t0_BB1	0	0	1	BB	1000	0,0%	0,0%	0,0%	0	0,43612	0
24BPF	t0_BB1	0	0	1	BB	1000	0,0%	0,0%	0,0%	0	0,43612	0
44BPF	t0_BB1	0	0	1	BB	1000	0,0%	0,0%	0,0%	0	0,43612	0
BPE	t0_BB1	0	0	1	BB	1000	0,0%	0,0%	0,0%	0	0,43612	0
BPA	t0_BB1	0	0	1	BB	1000	0,0%	0,0%	0,0%	0	0,43612	0
BPC	t0_BB1	0	0	1	BB	1000	0,0%	0,0%	0,0%	0	0,43612	0
BPB	t0_BB1	0	0	1	BB	1000	0,0%	0,0%	0,0%	0	0,43612	0

BP26DM	t0_BB1	0	0	1	BB	1000	0,0%	0,0%	0,0%	0	0,43612	0
BPC2	t0_BB1	0	0	1	BB	1000	0,0%	0,0%	0,0%	0	0,43612	0
BPZ	t0_BB1	0	0	1	BB	1000	0,0%	0,0%	0,0%	0	0,43612	0
BPS	t0_BB1	0	0	1	BB	1000	0,0%	0,0%	0,0%	0	0,43612	0
BPAP	t0_BB1	0	0	1	BB	1000	0,0%	0,0%	0,0%	0	0,43612	0
BPM	t0_BB1	0	0	1	BB	1000	0,0%	0,0%	0,0%	0	0,43612	0
BPP	t0_BB1	0	0	1	BB	1000	0,0%	0,0%	0,0%	0	0,43612	0
BPBP	t0_BB1	0	0	1	BB	1000	0,0%	0,0%	0,0%	0	0,43612	0
BPPH	t0_BB1	0	0	1	BB	1000	0,0%	0,0%	0,0%	0	0,43612	0
BPFL	t0_BB1	0	0	1	BB	1000	0,0%	0,0%	0,0%	0	0,43612	0
22BPF	t0_BB2	0	0	2	BB	1000	0,0%	0,0%	0,0%	0,0002	0,28913	0,000692
BPAF	t0_BB2	0	0	2	BB	1000	0,0%	0,0%	0,0%	0,0002	0,28913	0,000692
24BPF	t0_BB2	0	0	2	BB	1000	0,0%	0,0%	0,0%	0,0002	0,28913	0,000692
44BPF	t0_BB2	0	0	2	BB	1000	0,0%	0,0%	0,0%	0,0002	0,28913	0,000692
BPE	t0_BB2	0	0	2	BB	1000	0,0%	0,0%	0,0%	0,0002	0,28913	0,000692
BPA	t0_BB2	0	0	2	BB	1000	0,0%	0,0%	0,0%	0,0002	0,28913	0,000692
BPC	t0_BB2	0	0	2	BB	1000	0,0%	0,0%	0,0%	0,0002	0,28913	0,000692
BPB	t0_BB2	0	0	2	BB	1000	0,0%	0,0%	0,0%	0,0002	0,28913	0,000692
BP26DM	t0_BB2	0	0	2	BB	1000	0,0%	0,0%	0,0%	0,0002	0,28913	0,000692
BPC2	t0_BB2	0	0	2	BB	1000	0,0%	0,0%	0,0%	0,0002	0,28913	0,000692
BPZ	t0_BB2	0	0	2	BB	1000	0,0%	0,0%	0,0%	0,0002	0,28913	0,000692
BPS	t0_BB2	0	0	2	BB	1000	0,0%	0,0%	0,0%	0,0002	0,28913	0,000692
BPAP	t0_BB2	0	0	2	BB	1000	0,0%	0,0%	0,0%	0,0002	0,28913	0,000692
BPM	t0_BB2	0	0	2	BB	1000	0,0%	0,0%	0,0%	0,0002	0,28913	0,000692
BPP	t0_BB2	0	0	2	BB	1000	0,0%	0,0%	0,0%	0,0002	0,28913	0,000692
BPBP	t0_BB2	0	0	2	BB	1000	0,0%	0,0%	0,0%	0,0002	0,28913	0,000692
BPPH	t0_BB2	0	0	2	BB	1000	0,0%	0,0%	0,0%	0,0002	0,28913	0,000692
BPFL	t0_BB2	0	0	2	BB	1000	0,0%	0,0%	0,0%	0,0002	0,28913	0,000692
22BPF	t0_BB3	0	0	3	BB	1000	0,0%	0,0%	0,0%	0,00008	0,24136	0,000331
BPAF	t0_BB3	0	0	3	BB	1000	0,0%	0,0%	0,0%	0,00008	0,24136	0,000331
24BPF	t0_BB3	0	0	3	BB	1000	0,0%	0,0%	0,0%	0,00008	0,24136	0,000331
44BPF	t0_BB3	0	0	3	BB	1000	0,0%	0,0%	0,0%	0,00008	0,24136	0,000331
BPE	t0_BB3	0	0	3	BB	1000	0,0%	0,0%	0,0%	0,00008	0,24136	0,000331
BPA	t0_BB3	0	0	3	BB	1000	0,0%	0,0%	0,0%	0,00008	0,24136	0,000331
BPC	t0_BB3	0	0	3	BB	1000	0,0%	0,0%	0,0%	0,00008	0,24136	0,000331
BPB	t0_BB3	0	0	3	BB	1000	0,0%	0,0%	0,0%	0,00008	0,24136	0,000331
BP26DM	t0_BB3	0	0	3	BB	1000	0,0%	0,0%	0,0%	0,00008	0,24136	0,000331
BPC2	t0_BB3	0	0	3	BB	1000	0,0%	0,0%	0,0%	0,00008	0,24136	0,000331
BPZ	t0_BB3	0	0	3	BB	1000	0,0%	0,0%	0,0%	0,00008	0,24136	0,000331
BPS	t0_BB3	0	0	3	BB	1000	0,0%	0,0%	0,0%	0,00008	0,24136	0,000331
BPAP	t0_BB3	0	0	3	BB	1000	0,0%	0,0%	0,0%	0,00008	0,24136	0,000331
BPM	t0_BB3	0	0	3	BB	1000	0,0%	0,0%	0,0%	0,00008	0,24136	0,000331
BPP	t0_BB3	0	0	3	BB	1000	0,0%	0,0%	0,0%	0,00008	0,24136	0,000331
BPBP	t0_BB3	0	0	3	BB	1000	0,0%	0,0%	0,0%	0,00008	0,24136	0,000331
BPPH	t0_BB3	0	0	3	BB	1000	0,0%	0,0%	0,0%	0,00008	0,24136	0,000331
BPFL	t0_BB3	0	0	3	BB	1000	0,0%	0,0%	0,0%	0,00008	0,24136	0,000331
22BPF	t0_BK1	0	0	1	BK	1000	0,0%	0,0%	0,0%	0,98451	0,31888	3,0874
BPAF	t0_BK1	0	0	1	BK	1000	0,0%	0,0%	0,0%	0,98451	0,31888	3,0874
24BPF	t0_BK1	0	0	1	BK	1000	0,0%	0,0%	0,0%	0,98451	0,31888	3,0874
44BPF	t0_BK1	0	0	1	BK	1000	0,0%	0,0%	0,0%	0,98451	0,31888	3,0874
BPE	t0_BK1	0	0	1	BK	1000	0,0%	0,0%	0,0%	0,98451	0,31888	3,0874
BPA	t0_BK1	0	0	1	BK	1000	0,0%	0,0%	0,0%	0,98451	0,31888	3,0874
BPC	t0_BK1	0	0	1	BK	1000	0,0%	0,0%	0,0%	0,98451	0,31888	3,0874
BPB	t0_BK1	0	0	1	BK	1000	0,0%	0,0%	0,0%	0,98451	0,31888	3,0874
BP26DM	t0_BK1	0	0	1	BK	1000	0,0%	0,0%	0,0%	0,98451	0,31888	3,0874
BPC2	t0_BK1	0	0	1	BK	1000	0,0%	0,0%	0,0%	0,98451	0,31888	3,0874
BPZ	t0_BK1	0	0	1	BK	1000	0,0%	0,0%	0,0%	0,98451	0,31888	3,0874
BPS	t0_BK1	0	0	1	BK	1000	0,0%	0,0%	0,0%	0,98451	0,31888	3,0874
BPAP	t0_BK1	0	0	1	BK	1000	0,0%	0,0%	0,0%	0,98451	0,31888	3,0874
BPM	t0_BK1	0	0	1	BK	1000	0,0%	0,0%	0,0%	0,98451	0,31888	3,0874
BPP	t0_BK1	0	0	1	BK	1000	0,0%	0,0%	0,0%	0,98451	0,31888	3,0874
BPBP	t0_BK1	0	0	1	BK	1000	0,0%	0,0%	0,0%	0,98451	0,31888	3,0874
BPPH	t0_BK1	0	0	1	BK	1000	0,0%	0,0%	0,0%	0,98451	0,31888	3,0874
BPFL	t0_BK1	0	0	1	BK	1000	0,0%	0,0%	0,0%	0,98451	0,31888	3,0874
22BPF	t0_BK2	0	0	2	BK	1000	0,0%	0,0%	0,0%	0,97788	0,44535	2,195756
BPAF	t0_BK2	0	0	2	BK	1000	0,0%	0,0%	0,0%	0,97788	0,44535	2,195756
24BPF	t0_BK2	0	0	2	BK	1000	0,0%	0,0%	0,0%	0,97788	0,44535	2,195756
44BPF	t0_BK2	0	0	2	BK	1000	0,0%	0,0%	0,0%	0,97788	0,44535	2,195756
BPE	t0_BK2	0	0	2	BK	1000	0,0%	0,0%	0,0%	0,97788	0,44535	2,195756
BPA	t0_BK2	0	0	2	BK	1000	0,0%	0,0%	0,0%	0,97788	0,44535	2,195756
BPC	t0_BK2	0	0	2	BK	1000	0,0%	0,0%	0,0%	0,97788	0,44535	2,195756
BPB	t0_BK2	0	0	2	BK	1000	0,0%	0,0%	0,0%	0,97788	0,44535	2,195756
BP26DM	t0_BK2	0	0	2	BK	1000	0,0%	0,0%	0,0%	0,97788	0,44535	2,195756
BPC2	t0_BK2	0	0	2	BK	1000	0,0%	0,0%	0,0%	0,97788	0,44535	2,195756
BPZ	t0_BK2	0	0	2	BK	1000	0,0%	0,0%	0,0%	0,97788	0,44535	2,195756
BPS	t0_BK2	0	0	2	BK	1000	0,0%	0,0%	0,0%	0,97788	0,44535	2,195756
BPAP	t0_BK2	0	0	2	BK	1000	0,0%	0,0%	0,0%	0,97788	0,44535	2,195756
BPM	t0_BK2	0	0	2	BK	1000	0,0%	0,0%	0,0%	0,97788	0,44535	2,195756
BPP	t0_BK2	0	0	2	BK	1000	0,0%	0,0%	0,0%	0,97788	0,44535	2,195756
BPBP	t0_BK2	0	0	2	BK	1000	0,0%	0,0%	0,0%	0,97788	0,44535	2,195756
BPPH	t0_BK2	0	0	2	BK	1000	0,0%	0,0%	0,0%	0,97788	0,44535	2,195756
BPFL	t0_BK2	0	0	2	BK	1000	0,0%	0,0%	0,0%	0,97788	0,44535	2,195756
22BPF	t0_BK3	0	0	3	BK	1000	0,0%	0,0%	0,0%	0,95136	0,47011	2,023697
BPAF	t0_BK3	0	0	3	BK	1000	0,0%	0,0%	0,0%	0,95136	0,47011	2,023697
24BPF	t0_BK3	0	0	3	BK	1000	0,0%	0,0%	0,0%	0,95136	0,47011	2,023697
44BPF	t0_BK3	0	0	3	BK	1000	0,0%	0,0%	0,0%	0,95136	0,47011	2,023697

BPE	t0_BK3	0	0	3	BK	1000	0,0%	0,0%	0,0%	0,95136	0,47011	2,023697
BPA	t0_BK3	0	0	3	BK	1000	0,0%	0,0%	0,0%	0,95136	0,47011	2,023697
BPC	t0_BK3	0	0	3	BK	1000	0,0%	0,0%	0,0%	0,95136	0,47011	2,023697
BPB	t0_BK3	0	0	3	BK	1000	0,0%	0,0%	0,0%	0,95136	0,47011	2,023697
BP26DM	t0_BK3	0	0	3	BK	1000	0,0%	0,0%	0,0%	0,95136	0,47011	2,023697
BPC2	t0_BK3	0	0	3	BK	1000	0,0%	0,0%	0,0%	0,95136	0,47011	2,023697
BPZ	t0_BK3	0	0	3	BK	1000	0,0%	0,0%	0,0%	0,95136	0,47011	2,023697
BPS	t0_BK3	0	0	3	BK	1000	0,0%	0,0%	0,0%	0,95136	0,47011	2,023697
BPAP	t0_BK3	0	0	3	BK	1000	0,0%	0,0%	0,0%	0,95136	0,47011	2,023697
BPM	t0_BK3	0	0	3	BK	1000	0,0%	0,0%	0,0%	0,95136	0,47011	2,023697
BPP	t0_BK3	0	0	3	BK	1000	0,0%	0,0%	0,0%	0,95136	0,47011	2,023697
BPBP	t0_BK3	0	0	3	BK	1000	0,0%	0,0%	0,0%	0,95136	0,47011	2,023697
BPPH	t0_BK3	0	0	3	BK	1000	0,0%	0,0%	0,0%	0,95136	0,47011	2,023697
BPFL	t0_BK3	0	0	3	BK	1000	0,0%	0,0%	0,0%	0,95136	0,47011	2,023697
22BPF	t0_EA1	0	0	1	EA	1000	0,0%	0,0%	0,0%	1,00751	0,16217	6,212678
BPAF	t0_EA1	0	0	1	EA	1000	0,0%	0,0%	0,0%	1,00751	0,16217	6,212678
24BPF	t0_EA1	0	0	1	EA	1000	0,0%	0,0%	0,0%	1,00751	0,16217	6,212678
44BPF	t0_EA1	0	0	1	EA	1000	0,0%	0,0%	0,0%	1,00751	0,16217	6,212678
BPE	t0_EA1	0	0	1	EA	1000	0,0%	0,0%	0,0%	1,00751	0,16217	6,212678
BPA	t0_EA1	0	0	1	EA	1000	0,0%	0,0%	0,0%	1,00751	0,16217	6,212678
BPC	t0_EA1	0	0	1	EA	1000	0,0%	0,0%	0,0%	1,00751	0,16217	6,212678
BPB	t0_EA1	0	0	1	EA	1000	0,0%	0,0%	0,0%	1,00751	0,16217	6,212678
BP26DM	t0_EA1	0	0	1	EA	1000	0,0%	0,0%	0,0%	1,00751	0,16217	6,212678
BPC2	t0_EA1	0	0	1	EA	1000	0,0%	0,0%	0,0%	1,00751	0,16217	6,212678
BPZ	t0_EA1	0	0	1	EA	1000	0,0%	0,0%	0,0%	1,00751	0,16217	6,212678
BPS	t0_EA1	0	0	1	EA	1000	0,0%	0,0%	0,0%	1,00751	0,16217	6,212678
BPAP	t0_EA1	0	0	1	EA	1000	0,0%	0,0%	0,0%	1,00751	0,16217	6,212678
BPM	t0_EA1	0	0	1	EA	1000	0,0%	0,0%	0,0%	1,00751	0,16217	6,212678
BPP	t0_EA1	0	0	1	EA	1000	0,0%	0,0%	0,0%	1,00751	0,16217	6,212678
BPBP	t0_EA1	0	0	1	EA	1000	0,0%	0,0%	0,0%	1,00751	0,16217	6,212678
BPPH	t0_EA1	0	0	1	EA	1000	0,0%	0,0%	0,0%	1,00751	0,16217	6,212678
BPFL	t0_EA1	0	0	1	EA	1000	0,0%	0,0%	0,0%	1,00751	0,16217	6,212678
22BPF	t0_EA2	0	0	2	EA	1000	0,0%	0,0%	0,0%	1,02196	0,29124	3,508996
BPAF	t0_EA2	0	0	2	EA	1000	0,0%	0,0%	0,0%	1,02196	0,29124	3,508996
24BPF	t0_EA2	0	0	2	EA	1000	0,0%	0,0%	0,0%	1,02196	0,29124	3,508996
44BPF	t0_EA2	0	0	2	EA	1000	0,0%	0,0%	0,0%	1,02196	0,29124	3,508996
BPE	t0_EA2	0	0	2	EA	1000	0,0%	0,0%	0,0%	1,02196	0,29124	3,508996
BPA	t0_EA2	0	0	2	EA	1000	0,0%	0,0%	0,0%	1,02196	0,29124	3,508996
BPC	t0_EA2	0	0	2	EA	1000	0,0%	0,0%	0,0%	1,02196	0,29124	3,508996
BPB	t0_EA2	0	0	2	EA	1000	0,0%	0,0%	0,0%	1,02196	0,29124	3,508996
BP26DM	t0_EA2	0	0	2	EA	1000	0,0%	0,0%	0,0%	1,02196	0,29124	3,508996
BPC2	t0_EA2	0	0	2	EA	1000	0,0%	0,0%	0,0%	1,02196	0,29124	3,508996
BPZ	t0_EA2	0	0	2	EA	1000	0,0%	0,0%	0,0%	1,02196	0,29124	3,508996
BPS	t0_EA2	0	0	2	EA	1000	0,0%	0,0%	0,0%	1,02196	0,29124	3,508996
BPAP	t0_EA2	0	0	2	EA	1000	0,0%	0,0%	0,0%	1,02196	0,29124	3,508996
BPM	t0_EA2	0	0	2	EA	1000	0,0%	0,0%	0,0%	1,02196	0,29124	3,508996
BPP	t0_EA2	0	0	2	EA	1000	0,0%	0,0%	0,0%	1,02196	0,29124	3,508996
BPBP	t0_EA2	0	0	2	EA	1000	0,0%	0,0%	0,0%	1,02196	0,29124	3,508996
BPPH	t0_EA2	0	0	2	EA	1000	0,0%	0,0%	0,0%	1,02196	0,29124	3,508996
BPFL	t0_EA2	0	0	2	EA	1000	0,0%	0,0%	0,0%	1,02196	0,29124	3,508996
22BPF	t0_EA3	0	0	3	EA	1000	0,0%	0,0%	0,0%	1,02851	0,20103	5,116202
BPAF	t0_EA3	0	0	3	EA	1000	0,0%	0,0%	0,0%	1,02851	0,20103	5,116202
24BPF	t0_EA3	0	0	3	EA	1000	0,0%	0,0%	0,0%	1,02851	0,20103	5,116202
44BPF	t0_EA3	0	0	3	EA	1000	0,0%	0,0%	0,0%	1,02851	0,20103	5,116202
BPE	t0_EA3	0	0	3	EA	1000	0,0%	0,0%	0,0%	1,02851	0,20103	5,116202
BPA	t0_EA3	0	0	3	EA	1000	0,0%	0,0%	0,0%	1,02851	0,20103	5,116202
BPC	t0_EA3	0	0	3	EA	1000	0,0%	0,0%	0,0%	1,02851	0,20103	5,116202
BPB	t0_EA3	0	0	3	EA	1000	0,0%	0,0%	0,0%	1,02851	0,20103	5,116202
BP26DM	t0_EA3	0	0	3	EA	1000	0,0%	0,0%	0,0%	1,02851	0,20103	5,116202
BPC2	t0_EA3	0	0	3	EA	1000	0,0%	0,0%	0,0%	1,02851	0,20103	5,116202
BPZ	t0_EA3	0	0	3	EA	1000	0,0%	0,0%	0,0%	1,02851	0,20103	5,116202
BPS	t0_EA3	0	0	3	EA	1000	0,0%	0,0%	0,0%	1,02851	0,20103	5,116202
BPAP	t0_EA3	0	0	3	EA	1000	0,0%	0,0%	0,0%	1,02851	0,20103	5,116202
BPM	t0_EA3	0	0	3	EA	1000	0,0%	0,0%	0,0%	1,02851	0,20103	5,116202
BPP	t0_EA3	0	0	3	EA	1000	0,0%	0,0%	0,0%	1,02851	0,20103	5,116202
BPBP	t0_EA3	0	0	3	EA	1000	0,0%	0,0%	0,0%	1,02851	0,20103	5,116202
BPPH	t0_EA3	0	0	3	EA	1000	0,0%	0,0%	0,0%	1,02851	0,20103	5,116202
BPFL	t0_EA3	0	0	3	EA	1000	0,0%	0,0%	0,0%	1,02851	0,20103	5,116202
22BPF	t0_EB1	0	0	1	EB	1000	0,0%	0,0%	0,0%	0,00016	0,66419	0,000241
BPAF	t0_EB1	0	0	1	EB	1000	0,0%	0,0%	0,0%	0,00016	0,66419	0,000241
24BPF	t0_EB1	0	0	1	EB	1000	0,0%	0,0%	0,0%	0,00016	0,66419	0,000241
44BPF	t0_EB1	0	0	1	EB	1000	0,0%	0,0%	0,0%	0,00016	0,66419	0,000241
BPE	t0_EB1	0	0	1	EB	1000	0,0%	0,0%	0,0%	0,00016	0,66419	0,000241
BPA	t0_EB1	0	0	1	EB	1000	0,0%	0,0%	0,0%	0,00016	0,66419	0,000241
BPC	t0_EB1	0	0	1	EB	1000	0,0%	0,0%	0,0%	0,00016	0,66419	0,000241
BPB	t0_EB1	0	0	1	EB	1000	0,0%	0,0%	0,0%	0,00016	0,66419	0,000241
BP26DM	t0_EB1	0	0	1	EB	1000	0,0%	0,0%	0,0%	0,00016	0,66419	0,000241
BPC2	t0_EB1	0	0	1	EB	1000	0,0%	0,0%	0,0%	0,00016	0,66419	0,000241
BPZ	t0_EB1	0	0	1	EB	1000	0,0%	0,0%	0,0%	0,00016	0,66419	0,000241
BPS	t0_EB1	0	0	1	EB	1000	0,0%	0,0%	0,0%	0,00016	0,66419	0,000241
BPAP	t0_EB1	0	0	1	EB	1000	0,0%	0,0%	0,0%	0,00016	0,66419	0,000241
BPM	t0_EB1	0	0	1	EB	1000	0,0%	0,0%	0,0%	0,00016	0,66419	0,000241
BPP	t0_EB1	0	0	1	EB	1000	0,0%	0,0%	0,0%	0,00016	0,66419	0,000241
BPBP	t0_EB1	0	0	1	EB	1000	0,0%	0,0%	0,0%	0,00016	0,66419	0,000241
BPPH	t0_EB1	0	0	1	EB	1000	0,0%	0,0%	0,0%	0,00016	0,66419	0,000241
BPFL	t0_EB1	0	0	1	EB	1000	0,0%	0,0%	0,0%	0,00016	0,66419	0,000241

22BPF	t0_EB2	0	0	2	EB	1000	0,0%	0,0%	0,0%	0,00012	0,42938	0,000279
BPAF	t0_EB2	0	0	2	EB	1000	0,0%	0,0%	0,0%	0,00012	0,42938	0,000279
24BPF	t0_EB2	0	0	2	EB	1000	0,0%	0,0%	0,0%	0,00012	0,42938	0,000279
44BPF	t0_EB2	0	0	2	EB	1000	0,0%	0,0%	0,0%	0,00012	0,42938	0,000279
BPE	t0_EB2	0	0	2	EB	1000	0,0%	0,0%	0,0%	0,00012	0,42938	0,000279
BPA	t0_EB2	0	0	2	EB	1000	0,0%	0,0%	0,0%	0,00012	0,42938	0,000279
BPC	t0_EB2	0	0	2	EB	1000	0,0%	0,0%	0,0%	0,00012	0,42938	0,000279
BPB	t0_EB2	0	0	2	EB	1000	0,0%	0,0%	0,0%	0,00012	0,42938	0,000279
BP26DM	t0_EB2	0	0	2	EB	1000	0,0%	0,0%	0,0%	0,00012	0,42938	0,000279
BPC2	t0_EB2	0	0	2	EB	1000	0,0%	0,0%	0,0%	0,00012	0,42938	0,000279
BPZ	t0_EB2	0	0	2	EB	1000	0,0%	0,0%	0,0%	0,00012	0,42938	0,000279
BPS	t0_EB2	0	0	2	EB	1000	0,0%	0,0%	0,0%	0,00012	0,42938	0,000279
BPAP	t0_EB2	0	0	2	EB	1000	0,0%	0,0%	0,0%	0,00012	0,42938	0,000279
BPM	t0_EB2	0	0	2	EB	1000	0,0%	0,0%	0,0%	0,00012	0,42938	0,000279
BPP	t0_EB2	0	0	2	EB	1000	0,0%	0,0%	0,0%	0,00012	0,42938	0,000279
BPBP	t0_EB2	0	0	2	EB	1000	0,0%	0,0%	0,0%	0,00012	0,42938	0,000279
BPPH	t0_EB2	0	0	2	EB	1000	0,0%	0,0%	0,0%	0,00012	0,42938	0,000279
BPFL	t0_EB2	0	0	2	EB	1000	0,0%	0,0%	0,0%	0,00012	0,42938	0,000279
22BPF	t0_EB3	0	0	3	EB	1000	0,0%	0,0%	0,0%	0,00028	0,51067	0,000548
BPAF	t0_EB3	0	0	3	EB	1000	0,0%	0,0%	0,0%	0,00028	0,51067	0,000548
24BPF	t0_EB3	0	0	3	EB	1000	0,0%	0,0%	0,0%	0,00028	0,51067	0,000548
44BPF	t0_EB3	0	0	3	EB	1000	0,0%	0,0%	0,0%	0,00028	0,51067	0,000548
BPE	t0_EB3	0	0	3	EB	1000	0,0%	0,0%	0,0%	0,00028	0,51067	0,000548
BPA	t0_EB3	0	0	3	EB	1000	0,0%	0,0%	0,0%	0,00028	0,51067	0,000548
BPC	t0_EB3	0	0	3	EB	1000	0,0%	0,0%	0,0%	0,00028	0,51067	0,000548
BPB	t0_EB3	0	0	3	EB	1000	0,0%	0,0%	0,0%	0,00028	0,51067	0,000548
BP26DM	t0_EB3	0	0	3	EB	1000	0,0%	0,0%	0,0%	0,00028	0,51067	0,000548
BPC2	t0_EB3	0	0	3	EB	1000	0,0%	0,0%	0,0%	0,00028	0,51067	0,000548
BPZ	t0_EB3	0	0	3	EB	1000	0,0%	0,0%	0,0%	0,00028	0,51067	0,000548
BPS	t0_EB3	0	0	3	EB	1000	0,0%	0,0%	0,0%	0,00028	0,51067	0,000548
BPAP	t0_EB3	0	0	3	EB	1000	0,0%	0,0%	0,0%	0,00028	0,51067	0,000548
BPM	t0_EB3	0	0	3	EB	1000	0,0%	0,0%	0,0%	0,00028	0,51067	0,000548
BPP	t0_EB3	0	0	3	EB	1000	0,0%	0,0%	0,0%	0,00028	0,51067	0,000548
BPBP	t0_EB3	0	0	3	EB	1000	0,0%	0,0%	0,0%	0,00028	0,51067	0,000548
BPPH	t0_EB3	0	0	3	EB	1000	0,0%	0,0%	0,0%	0,00028	0,51067	0,000548
BPFL	t0_EB3	0	0	3	EB	1000	0,0%	0,0%	0,0%	0,00028	0,51067	0,000548
22BPF	t0_EK1	0	0	1	EK	1000	0,0%	0,0%	0,0%	1,00036	0,64235	1,557344
BPAF	t0_EK1	0	0	1	EK	1000	0,0%	0,0%	0,0%	1,00036	0,64235	1,557344
24BPF	t0_EK1	0	0	1	EK	1000	0,0%	0,0%	0,0%	1,00036	0,64235	1,557344
44BPF	t0_EK1	0	0	1	EK	1000	0,0%	0,0%	0,0%	1,00036	0,64235	1,557344
BPE	t0_EK1	0	0	1	EK	1000	0,0%	0,0%	0,0%	1,00036	0,64235	1,557344
BPA	t0_EK1	0	0	1	EK	1000	0,0%	0,0%	0,0%	1,00036	0,64235	1,557344
BPC	t0_EK1	0	0	1	EK	1000	0,0%	0,0%	0,0%	1,00036	0,64235	1,557344
BPB	t0_EK1	0	0	1	EK	1000	0,0%	0,0%	0,0%	1,00036	0,64235	1,557344
BP26DM	t0_EK1	0	0	1	EK	1000	0,0%	0,0%	0,0%	1,00036	0,64235	1,557344
BPC2	t0_EK1	0	0	1	EK	1000	0,0%	0,0%	0,0%	1,00036	0,64235	1,557344
BPZ	t0_EK1	0	0	1	EK	1000	0,0%	0,0%	0,0%	1,00036	0,64235	1,557344
BPS	t0_EK1	0	0	1	EK	1000	0,0%	0,0%	0,0%	1,00036	0,64235	1,557344
BPAP	t0_EK1	0	0	1	EK	1000	0,0%	0,0%	0,0%	1,00036	0,64235	1,557344
BPM	t0_EK1	0	0	1	EK	1000	0,0%	0,0%	0,0%	1,00036	0,64235	1,557344
BPP	t0_EK1	0	0	1	EK	1000	0,0%	0,0%	0,0%	1,00036	0,64235	1,557344
BPBP	t0_EK1	0	0	1	EK	1000	0,0%	0,0%	0,0%	1,00036	0,64235	1,557344
BPPH	t0_EK1	0	0	1	EK	1000	0,0%	0,0%	0,0%	1,00036	0,64235	1,557344
BPFL	t0_EK1	0	0	1	EK	1000	0,0%	0,0%	0,0%	1,00036	0,64235	1,557344
22BPF	t0_EK2	0	0	2	EK	1000	0,0%	0,0%	0,0%	0,97488	0,43928	2,219268
BPAF	t0_EK2	0	0	2	EK	1000	0,0%	0,0%	0,0%	0,97488	0,43928	2,219268
24BPF	t0_EK2	0	0	2	EK	1000	0,0%	0,0%	0,0%	0,97488	0,43928	2,219268
44BPF	t0_EK2	0	0	2	EK	1000	0,0%	0,0%	0,0%	0,97488	0,43928	2,219268
BPE	t0_EK2	0	0	2	EK	1000	0,0%	0,0%	0,0%	0,97488	0,43928	2,219268
BPA	t0_EK2	0	0	2	EK	1000	0,0%	0,0%	0,0%	0,97488	0,43928	2,219268
BPC	t0_EK2	0	0	2	EK	1000	0,0%	0,0%	0,0%	0,97488	0,43928	2,219268
BPB	t0_EK2	0	0	2	EK	1000	0,0%	0,0%	0,0%	0,97488	0,43928	2,219268
BP26DM	t0_EK2	0	0	2	EK	1000	0,0%	0,0%	0,0%	0,97488	0,43928	2,219268
BPC2	t0_EK2	0	0	2	EK	1000	0,0%	0,0%	0,0%	0,97488	0,43928	2,219268
BPZ	t0_EK2	0	0	2	EK	1000	0,0%	0,0%	0,0%	0,97488	0,43928	2,219268
BPS	t0_EK2	0	0	2	EK	1000	0,0%	0,0%	0,0%	0,97488	0,43928	2,219268
BPAP	t0_EK2	0	0	2	EK	1000	0,0%	0,0%	0,0%	0,97488	0,43928	2,219268
BPM	t0_EK2	0	0	2	EK	1000	0,0%	0,0%	0,0%	0,97488	0,43928	2,219268
BPP	t0_EK2	0	0	2	EK	1000	0,0%	0,0%	0,0%	0,97488	0,43928	2,219268
BPBP	t0_EK2	0	0	2	EK	1000	0,0%	0,0%	0,0%	0,97488	0,43928	2,219268
BPPH	t0_EK2	0	0	2	EK	1000	0,0%	0,0%	0,0%	0,97488	0,43928	2,219268
BPFL	t0_EK2	0	0	2	EK	1000	0,0%	0,0%	0,0%	0,97488	0,43928	2,219268
22BPF	t0_EK3	0	0	3	EK	1000	0,0%	0,0%	0,0%	1,00555	0,50885	1,976123
BPAF	t0_EK3	0	0	3	EK	1000	0,0%	0,0%	0,0%	1,00555	0,50885	1,976123
24BPF	t0_EK3	0	0	3	EK	1000	0,0%	0,0%	0,0%	1,00555	0,50885	1,976123
44BPF	t0_EK3	0	0	3	EK	1000	0,0%	0,0%	0,0%	1,00555	0,50885	1,976123
BPE	t0_EK3	0	0	3	EK	1000	0,0%	0,0%	0,0%	1,00555	0,50885	1,976123
BPA	t0_EK3	0	0	3	EK	1000	0,0%	0,0%	0,0%	1,00555	0,50885	1,976123
BPC	t0_EK3	0	0	3	EK	1000	0,0%	0,0%	0,0%	1,00555	0,50885	1,976123
BPB	t0_EK3	0	0	3	EK	1000	0,0%	0,0%	0,0%	1,00555	0,50885	1,976123
BP26DM	t0_EK3	0	0	3	EK	1000	0,0%	0,0%	0,0%	1,00555	0,50885	1,976123
BPC2	t0_EK3	0	0	3	EK	1000	0,0%	0,0%	0,0%	1,00555	0,50885	1,976123
BPZ	t0_EK3	0	0	3	EK	1000	0,0%	0,0%	0,0%	1,00555	0,50885	1,976123
BPS	t0_EK3	0	0	3	EK	1000	0,0%	0,0%	0,0%	1,00555	0,50885	1,976123
BPAP	t0_EK3	0	0	3	EK	1000	0,0%	0,0%	0,0%	1,00555	0,50885	1,976123
BPM	t0_EK3	0	0	3	EK	1000	0,0%	0,0%	0,0%	1,00555	0,50885	1,976123

BPP	t0_EK3	0	0	3	EK	1000	0,0%	0,0%	0,0%	1,00555	0,50885	1,976123
BPBP	t0_EK3	0	0	3	EK	1000	0,0%	0,0%	0,0%	1,00555	0,50885	1,976123
BPPH	t0_EK3	0	0	3	EK	1000	0,0%	0,0%	0,0%	1,00555	0,50885	1,976123
BPFL	t0_EK3	0	0	3	EK	1000	0,0%	0,0%	0,0%	1,00555	0,50885	1,976123
22BPF	t3_ABS1	3	72	1	ABS	1032,18614	-3,2%	-3,2%	0,0%	0	0,034	0
BPAF	t3_ABS1	3	72	1	ABS	913,694213	8,6%	8,6%	0,0%	0	0,034	0
24BPF	t3_ABS1	3	72	1	ABS	1037,73318	-3,8%	-3,8%	0,0%	0	0,034	0
44BPF	t3_ABS1	3	72	1	ABS	879,768231	12,0%	12,0%	0,0%	0	0,034	0
BPE	t3_ABS1	3	72	1	ABS	885,206522	11,5%	11,5%	0,0%	0	0,034	0
BPA	t3_ABS1	3	72	1	ABS	828,731549	17,1%	17,1%	0,0%	0	0,034	0
BPC	t3_ABS1	3	72	1	ABS	829,904751	17,0%	17,0%	0,0%	0	0,034	0
BPB	t3_ABS1	3	72	1	ABS	882,012963	11,8%	11,8%	0,0%	0	0,034	0
BP26DM	t3_ABS1	3	72	1	ABS	679,017373	32,1%	32,1%	0,0%	0	0,034	0
BPC2	t3_ABS1	3	72	1	ABS	767,694845	23,2%	23,2%	0,0%	0	0,034	0
BPZ	t3_ABS1	3	72	1	ABS	1064,55535	-6,5%	-6,5%	0,0%	0	0,034	0
BPS	t3_ABS1	3	72	1	ABS	839,808876	16,0%	16,0%	0,0%	0	0,034	0
BPAP	t3_ABS1	3	72	1	ABS	1017,16407	-1,7%	-1,7%	0,0%	0	0,034	0
BPM	t3_ABS1	3	72	1	ABS	460,183959	54,0%	54,0%	0,0%	0	0,034	0
BPP	t3_ABS1	3	72	1	ABS	323,676398	67,6%	67,6%	0,0%	0	0,034	0
BPBP	t3_ABS1	3	72	1	ABS	534,543084	46,5%	46,5%	0,0%	0	0,034	0
BPPH	t3_ABS1	3	72	1	ABS	142,911919	85,7%	85,7%	0,0%	0	0,034	0
BPFL	t3_ABS1	3	72	1	ABS	508,18943	49,2%	49,2%	0,0%	0	0,034	0
22BPF	t3_ABS2	3	72	2	ABS	801,506669	19,8%	19,8%	0,0%	0,001	0,063	0,015873
BPAF	t3_ABS2	3	72	2	ABS	812,072437	18,8%	18,8%	0,0%	0,001	0,063	0,015873
24BPF	t3_ABS2	3	72	2	ABS	784,813498	21,5%	21,5%	0,0%	0,001	0,063	0,015873
44BPF	t3_ABS2	3	72	2	ABS	808,702924	19,1%	19,1%	0,0%	0,001	0,063	0,015873
BPE	t3_ABS2	3	72	2	ABS	694,044442	30,6%	30,6%	0,0%	0,001	0,063	0,015873
BPA	t3_ABS2	3	72	2	ABS	856,620946	14,3%	14,3%	0,0%	0,001	0,063	0,015873
BPC	t3_ABS2	3	72	2	ABS	796,091741	20,4%	20,4%	0,0%	0,001	0,063	0,015873
BPB	t3_ABS2	3	72	2	ABS	803,489942	19,7%	19,7%	0,0%	0,001	0,063	0,015873
BP26DM	t3_ABS2	3	72	2	ABS	702,406275	29,8%	29,8%	0,0%	0,001	0,063	0,015873
BPC2	t3_ABS2	3	72	2	ABS	712,921175	28,7%	28,7%	0,0%	0,001	0,063	0,015873
BPZ	t3_ABS2	3	72	2	ABS	793,994873	20,6%	20,6%	0,0%	0,001	0,063	0,015873
BPS	t3_ABS2	3	72	2	ABS	798,866812	20,1%	20,1%	0,0%	0,001	0,063	0,015873
BPAP	t3_ABS2	3	72	2	ABS	762,564922	23,7%	23,7%	0,0%	0,001	0,063	0,015873
BPM	t3_ABS2	3	72	2	ABS	369,684849	63,0%	63,0%	0,0%	0,001	0,063	0,015873
BPP	t3_ABS2	3	72	2	ABS	241,048793	75,9%	75,9%	0,0%	0,001	0,063	0,015873
BPBP	t3_ABS2	3	72	2	ABS	428,405579	57,2%	57,2%	0,0%	0,001	0,063	0,015873
BPPH	t3_ABS2	3	72	2	ABS	91,0362056	90,9%	90,9%	0,0%	0,001	0,063	0,015873
BPFL	t3_ABS2	3	72	2	ABS	465,492998	53,5%	53,5%	0,0%	0,001	0,063	0,015873
22BPF	t3_ABS3	3	72	3	ABS	748,586497	25,1%	25,1%	0,0%	0	0,03	0
BPAF	t3_ABS3	3	72	3	ABS	680,994889	31,9%	31,9%	0,0%	0	0,03	0
24BPF	t3_ABS3	3	72	3	ABS	681,199678	31,9%	31,9%	0,0%	0	0,03	0
44BPF	t3_ABS3	3	72	3	ABS	783,988098	21,6%	21,6%	0,0%	0	0,03	0
BPE	t3_ABS3	3	72	3	ABS	718,177485	28,2%	28,2%	0,0%	0	0,03	0
BPA	t3_ABS3	3	72	3	ABS	874,213516	12,6%	12,6%	0,0%	0	0,03	0
BPC	t3_ABS3	3	72	3	ABS	753,889916	24,6%	24,6%	0,0%	0	0,03	0
BPB	t3_ABS3	3	72	3	ABS	767,410514	23,3%	23,3%	0,0%	0	0,03	0
BP26DM	t3_ABS3	3	72	3	ABS	688,179098	31,2%	31,2%	0,0%	0	0,03	0
BPC2	t3_ABS3	3	72	3	ABS	679,208205	32,1%	32,1%	0,0%	0	0,03	0
BPZ	t3_ABS3	3	72	3	ABS	779,431349	22,1%	22,1%	0,0%	0	0,03	0
BPS	t3_ABS3	3	72	3	ABS	755,991738	24,4%	24,4%	0,0%	0	0,03	0
BPAP	t3_ABS3	3	72	3	ABS	741,352533	25,9%	25,9%	0,0%	0	0,03	0
BPM	t3_ABS3	3	72	3	ABS	421,102998	57,9%	57,9%	0,0%	0	0,03	0
BPP	t3_ABS3	3	72	3	ABS	314,075147	68,6%	68,6%	0,0%	0	0,03	0
BPBP	t3_ABS3	3	72	3	ABS	458,908215	54,1%	54,1%	0,0%	0	0,03	0
BPPH	t3_ABS3	3	72	3	ABS	178,263569	82,2%	82,2%	0,0%	0	0,03	0
BPFL	t3_ABS3	3	72	3	ABS	421,39684	57,9%	57,9%	0,0%	0	0,03	0
22BPF	t3_ABT1	3	72	1	ABT	945,047862	5,5%	5,5%	0,0%	0,001	0,053	0,018868
BPAF	t3_ABT1	3	72	1	ABT	841,025229	15,9%	15,9%	0,0%	0,001	0,053	0,018868
24BPF	t3_ABT1	3	72	1	ABT	891,235949	10,9%	10,9%	0,0%	0,001	0,053	0,018868
44BPF	t3_ABT1	3	72	1	ABT	811,76429	18,8%	18,8%	0,0%	0,001	0,053	0,018868
BPE	t3_ABT1	3	72	1	ABT	806,057157	19,4%	19,4%	0,0%	0,001	0,053	0,018868
BPA	t3_ABT1	3	72	1	ABT	776,923147	22,3%	22,3%	0,0%	0,001	0,053	0,018868
BPC	t3_ABT1	3	72	1	ABT	747,618865	25,2%	25,2%	0,0%	0,001	0,053	0,018868
BPB	t3_ABT1	3	72	1	ABT	800,391538	20,0%	20,0%	0,0%	0,001	0,053	0,018868
BP26DM	t3_ABT1	3	72	1	ABT	681,699048	31,8%	31,8%	0,0%	0,001	0,053	0,018868
BPC2	t3_ABT1	3	72	1	ABT	733,138464	26,7%	26,7%	0,0%	0,001	0,053	0,018868
BPZ	t3_ABT1	3	72	1	ABT	778,245719	22,2%	22,2%	0,0%	0,001	0,053	0,018868
BPS	t3_ABT1	3	72	1	ABT	810,781103	18,9%	18,9%	0,0%	0,001	0,053	0,018868
BPAP	t3_ABT1	3	72	1	ABT	756,023602	24,4%	24,4%	0,0%	0,001	0,053	0,018868
BPM	t3_ABT1	3	72	1	ABT	373,348182	62,7%	62,7%	0,0%	0,001	0,053	0,018868
BPP	t3_ABT1	3	72	1	ABT	337,548632	66,2%	66,2%	0,0%	0,001	0,053	0,018868
BPBP	t3_ABT1	3	72	1	ABT	555,24421	44,5%	44,5%	0,0%	0,001	0,053	0,018868
BPPH	t3_ABT1	3	72	1	ABT	137,225904	86,3%	86,3%	0,0%	0,001	0,053	0,018868
BPFL	t3_ABT1	3	72	1	ABT	497,170633	50,3%	50,3%	0,0%	0,001	0,053	0,018868
22BPF	t3_ABT2	3	72	2	ABT	796,016853	20,4%	20,4%	0,0%	0	0,046	0
BPAF	t3_ABT2	3	72	2	ABT	673,009085	32,7%	32,7%	0,0%	0	0,046	0
24BPF	t3_ABT2	3	72	2	ABT	786,060366	21,4%	21,4%	0,0%	0	0,046	0
44BPF	t3_ABT2	3	72	2	ABT	830,664717	16,9%	16,9%	0,0%	0	0,046	0
BPE	t3_ABT2	3	72	2	ABT	794,691559	20,5%	20,5%	0,0%	0	0,046	0
BPA	t3_ABT2	3	72	2	ABT	806,882865	19,3%	19,3%	0,0%	0	0,046	0
BPC	t3_ABT2	3	72	2	ABT	746,002346	25,4%	25,4%	0,0%	0	0,046	0
BPB	t3_ABT2	3	72	2	ABT	804,855092	19,5%	19,5%	0,0%	0	0,046	0
BP26DM	t3_ABT2	3	72	2	ABT	616,470793	38,4%	38,4%	0,0%	0	0,046	0
BPC2	t3_ABT2	3	72	2	ABT	760,029278	24,0%	24,0%	0,0%	0	0,046	0

BPZ	t3_ABT2	3	72	2	ABT	781,09527	21,9%	21,9%	0,0%	0	0,046	0
BPS	t3_ABT2	3	72	2	ABT	827,963536	17,2%	17,2%	0,0%	0	0,046	0
BPAP	t3_ABT2	3	72	2	ABT	763,525525	23,6%	23,6%	0,0%	0	0,046	0
BPM	t3_ABT2	3	72	2	ABT	395,040702	60,5%	60,5%	0,0%	0	0,046	0
BPP	t3_ABT2	3	72	2	ABT	301,018502	69,9%	69,9%	0,0%	0	0,046	0
BPBP	t3_ABT2	3	72	2	ABT	490,806787	50,9%	50,9%	0,0%	0	0,046	0
BPPH	t3_ABT2	3	72	2	ABT	149,467673	85,1%	85,1%	0,0%	0	0,046	0
BPFL	t3_ABT2	3	72	2	ABT	491,790182	50,8%	50,8%	0,0%	0	0,046	0
22BPF	t3_ABT3	3	72	3	ABT	970,457937	3,0%	3,0%	0,0%	0	0,054	0
BPAF	t3_ABT3	3	72	3	ABT	835,382988	16,5%	16,5%	0,0%	0	0,054	0
24BPF	t3_ABT3	3	72	3	ABT	943,821786	5,6%	5,6%	0,0%	0	0,054	0
44BPF	t3_ABT3	3	72	3	ABT	790,826962	20,9%	20,9%	0,0%	0	0,054	0
BPE	t3_ABT3	3	72	3	ABT	794,070328	20,6%	20,6%	0,0%	0	0,054	0
BPA	t3_ABT3	3	72	3	ABT	747,074553	25,3%	25,3%	0,0%	0	0,054	0
BPC	t3_ABT3	3	72	3	ABT	748,426751	25,2%	25,2%	0,0%	0	0,054	0
BPB	t3_ABT3	3	72	3	ABT	776,87516	22,3%	22,3%	0,0%	0	0,054	0
BP26DM	t3_ABT3	3	72	3	ABT	630,4753	37,0%	37,0%	0,0%	0	0,054	0
BPC2	t3_ABT3	3	72	3	ABT	729,674163	27,0%	27,0%	0,0%	0	0,054	0
BPZ	t3_ABT3	3	72	3	ABT	757,706205	24,2%	24,2%	0,0%	0	0,054	0
BPS	t3_ABT3	3	72	3	ABT	795,400597	20,5%	20,5%	0,0%	0	0,054	0
BPAP	t3_ABT3	3	72	3	ABT	732,955302	26,7%	26,7%	0,0%	0	0,054	0
BPM	t3_ABT3	3	72	3	ABT	421,474694	57,9%	57,9%	0,0%	0	0,054	0
BPP	t3_ABT3	3	72	3	ABT	389,60119	61,0%	61,0%	0,0%	0	0,054	0
BPBP	t3_ABT3	3	72	3	ABT	570,170243	43,0%	43,0%	0,0%	0	0,054	0
BPPH	t3_ABT3	3	72	3	ABT	217,74787	78,2%	78,2%	0,0%	0	0,054	0
BPFL	t3_ABT3	3	72	3	ABT	532,528151	46,7%	46,7%	0,0%	0	0,054	0
22BPF	t3_BA1	3	72	1	BA	1,56707076	99,8%	99,8%	0,0%	1,459	0,301	4,847176
BPAF	t3_BA1	3	72	1	BA	1,56024001	99,8%	99,8%	0,0%	1,459	0,301	4,847176
24BPF	t3_BA1	3	72	1	BA	0,72415293	99,9%	99,9%	0,0%	1,459	0,301	4,847176
44BPF	t3_BA1	3	72	1	BA	0,24758326	100,0%	100,0%	0,0%	1,459	0,301	4,847176
BPE	t3_BA1	3	72	1	BA	0,36801583	100,0%	100,0%	0,0%	1,459	0,301	4,847176
BPA	t3_BA1	3	72	1	BA	98,1052651	90,2%	90,2%	0,0%	1,459	0,301	4,847176
BPC	t3_BA1	3	72	1	BA	0,16976512	100,0%	100,0%	0,0%	1,459	0,301	4,847176
BPB	t3_BA1	3	72	1	BA	0,15682209	100,0%	100,0%	0,0%	1,459	0,301	4,847176
BP26DM	t3_BA1	3	72	1	BA	0,02721666	100,0%	100,0%	0,0%	1,459	0,301	4,847176
BPC2	t3_BA1	3	72	1	BA	0,1961482	100,0%	100,0%	0,0%	1,459	0,301	4,847176
BPZ	t3_BA1	3	72	1	BA	0,20147909	100,0%	100,0%	0,0%	1,459	0,301	4,847176
BPS	t3_BA1	3	72	1	BA	7,46885774	99,3%	99,3%	0,0%	1,459	0,301	4,847176
BPAP	t3_BA1	3	72	1	BA	0,2255244	100,0%	100,0%	0,0%	1,459	0,301	4,847176
BPM	t3_BA1	3	72	1	BA	0,04715453	100,0%	100,0%	0,0%	1,459	0,301	4,847176
BPP	t3_BA1	3	72	1	BA	0,98130319	99,9%	99,9%	0,0%	1,459	0,301	4,847176
BPBP	t3_BA1	3	72	1	BA	0,38503504	100,0%	100,0%	0,0%	1,459	0,301	4,847176
BPPH	t3_BA1	3	72	1	BA	0,0357408	100,0%	100,0%	0,0%	1,459	0,301	4,847176
BPFL	t3_BA1	3	72	1	BA	0,72415855	99,9%	99,9%	0,0%	1,459	0,301	4,847176
22BPF	t3_BA2	3	72	2	BA	0,25729448	100,0%	100,0%	0,0%	2,115	0,191	11,0733
BPAF	t3_BA2	3	72	2	BA	1,02328781	99,9%	99,9%	0,0%	2,115	0,191	11,0733
24BPF	t3_BA2	3	72	2	BA	0,59141416	99,9%	99,9%	0,0%	2,115	0,191	11,0733
44BPF	t3_BA2	3	72	2	BA	0,22063779	100,0%	100,0%	0,0%	2,115	0,191	11,0733
BPE	t3_BA2	3	72	2	BA	0,38267063	100,0%	100,0%	0,0%	2,115	0,191	11,0733
BPA	t3_BA2	3	72	2	BA	0	100,0%	100,0%	0,0%	2,115	0,191	11,0733
BPC	t3_BA2	3	72	2	BA	0,02321317	100,0%	100,0%	0,0%	2,115	0,191	11,0733
BPB	t3_BA2	3	72	2	BA	0,11683125	100,0%	100,0%	0,0%	2,115	0,191	11,0733
BP26DM	t3_BA2	3	72	2	BA	0,15530261	100,0%	100,0%	0,0%	2,115	0,191	11,0733
BPC2	t3_BA2	3	72	2	BA	0,06922178	100,0%	100,0%	0,0%	2,115	0,191	11,0733
BPZ	t3_BA2	3	72	2	BA	0,211622	100,0%	100,0%	0,0%	2,115	0,191	11,0733
BPS	t3_BA2	3	72	2	BA	2,30313457	99,8%	99,8%	0,0%	2,115	0,191	11,0733
BPAP	t3_BA2	3	72	2	BA	0,24516095	100,0%	100,0%	0,0%	2,115	0,191	11,0733
BPM	t3_BA2	3	72	2	BA	0,02050029	100,0%	100,0%	0,0%	2,115	0,191	11,0733
BPP	t3_BA2	3	72	2	BA	0,77109941	99,9%	99,9%	0,0%	2,115	0,191	11,0733
BPBP	t3_BA2	3	72	2	BA	0,05633084	100,0%	100,0%	0,0%	2,115	0,191	11,0733
BPPH	t3_BA2	3	72	2	BA	0,1327564	100,0%	100,0%	0,0%	2,115	0,191	11,0733
BPFL	t3_BA2	3	72	2	BA	0,44440798	100,0%	100,0%	0,0%	2,115	0,191	11,0733
22BPF	t3_BA3	3	72	3	BA	0,40168251	100,0%	100,0%	0,0%	2,47	0,167	14,79042
BPAF	t3_BA3	3	72	3	BA	0,81010747	99,9%	99,9%	0,0%	2,47	0,167	14,79042
24BPF	t3_BA3	3	72	3	BA	0,5171348	99,9%	99,9%	0,0%	2,47	0,167	14,79042
44BPF	t3_BA3	3	72	3	BA	0,89913077	99,9%	99,9%	0,0%	2,47	0,167	14,79042
BPE	t3_BA3	3	72	3	BA	0,30060848	100,0%	100,0%	0,0%	2,47	0,167	14,79042
BPA	t3_BA3	3	72	3	BA	9,49412452	99,1%	99,1%	0,0%	2,47	0,167	14,79042
BPC	t3_BA3	3	72	3	BA	0,01502825	100,0%	100,0%	0,0%	2,47	0,167	14,79042
BPB	t3_BA3	3	72	3	BA	0,04485967	100,0%	100,0%	0,0%	2,47	0,167	14,79042
BP26DM	t3_BA3	3	72	3	BA	0,20622278	100,0%	100,0%	0,0%	2,47	0,167	14,79042
BPC2	t3_BA3	3	72	3	BA	0,13710727	100,0%	100,0%	0,0%	2,47	0,167	14,79042
BPZ	t3_BA3	3	72	3	BA	0,04746308	100,0%	100,0%	0,0%	2,47	0,167	14,79042
BPS	t3_BA3	3	72	3	BA	1,93193506	99,8%	99,8%	0,0%	2,47	0,167	14,79042
BPAP	t3_BA3	3	72	3	BA	0,05871209	100,0%	100,0%	0,0%	2,47	0,167	14,79042
BPM	t3_BA3	3	72	3	BA	0,02381096	100,0%	100,0%	0,0%	2,47	0,167	14,79042
BPP	t3_BA3	3	72	3	BA	0,599321	99,9%	99,9%	0,0%	2,47	0,167	14,79042
BPBP	t3_BA3	3	72	3	BA	0,09041522	100,0%	100,0%	0,0%	2,47	0,167	14,79042
BPPH	t3_BA3	3	72	3	BA	0,02090573	100,0%	100,0%	0,0%	2,47	0,167	14,79042
BPFL	t3_BA3	3	72	3	BA	0,18450133	100,0%	100,0%	0,0%	2,47	0,167	14,79042
22BPF	t3_BB1	3	72	1	BB	1,4947617	99,9%	99,9%	0,0%	0	9,873	0
BPAF	t3_BB1	3	72	1	BB	1,89935113	99,8%	99,8%	0,0%	0	9,873	0
24BPF	t3_BB1	3	72	1	BB	1,13066908	99,9%	99,9%	0,0%	0	9,873	0
44BPF	t3_BB1	3	72	1	BB	1,07156419	99,9%	99,9%	0,0%	0	9,873	0
BPE	t3_BB1	3	72	1	BB	0,39306027	100,0%	100,0%	0,0%	0	9,873	0
BPA	t3_BB1	3	72	1	BB	0	100,0%	100,0%	0,0%	0	9,873	0

BPC	t3_BB1	3	72	1	BB	0,10028078	100,0%	100,0%	0,0%	0	9,873	0
BPB	t3_BB1	3	72	1	BB	0,51096207	99,9%	99,9%	0,0%	0	9,873	0
BP26DM	t3_BB1	3	72	1	BB	0,25763786	100,0%	100,0%	0,0%	0	9,873	0
BPC2	t3_BB1	3	72	1	BB	0,77625469	99,9%	99,9%	0,0%	0	9,873	0
BPZ	t3_BB1	3	72	1	BB	0,13810709	100,0%	100,0%	0,0%	0	9,873	0
BPS	t3_BB1	3	72	1	BB	5,70464337	99,4%	99,4%	0,0%	0	9,873	0
BPAP	t3_BB1	3	72	1	BB	0,29274244	100,0%	100,0%	0,0%	0	9,873	0
BPM	t3_BB1	3	72	1	BB	0,06516597	100,0%	100,0%	0,0%	0	9,873	0
BPP	t3_BB1	3	72	1	BB	0,68279672	99,9%	99,9%	0,0%	0	9,873	0
BPBP	t3_BB1	3	72	1	BB	0,12664242	100,0%	100,0%	0,0%	0	9,873	0
BPPH	t3_BB1	3	72	1	BB	0,11650976	100,0%	100,0%	0,0%	0	9,873	0
BPFL	t3_BB1	3	72	1	BB	0,27592375	100,0%	100,0%	0,0%	0	9,873	0
22BPF	t3_BB2	3	72	2	BB	0,62179964	99,9%	99,9%	0,0%	0	10,077	0
BPAF	t3_BB2	3	72	2	BB	1,18784574	99,9%	99,9%	0,0%	0	10,077	0
24BPF	t3_BB2	3	72	2	BB	0,83074567	99,9%	99,9%	0,0%	0	10,077	0
44BPF	t3_BB2	3	72	2	BB	0,72337904	99,9%	99,9%	0,0%	0	10,077	0
BPE	t3_BB2	3	72	2	BB	0,14830391	100,0%	100,0%	0,0%	0	10,077	0
BPA	t3_BB2	3	72	2	BB	0	100,0%	100,0%	0,0%	0	10,077	0
BPC	t3_BB2	3	72	2	BB	0,15747908	100,0%	100,0%	0,0%	0	10,077	0
BPB	t3_BB2	3	72	2	BB	0,05810414	100,0%	100,0%	0,0%	0	10,077	0
BP26DM	t3_BB2	3	72	2	BB	0,2112777	100,0%	100,0%	0,0%	0	10,077	0
BPC2	t3_BB2	3	72	2	BB	0,14319836	100,0%	100,0%	0,0%	0	10,077	0
BPZ	t3_BB2	3	72	2	BB	0,09083223	100,0%	100,0%	0,0%	0	10,077	0
BPS	t3_BB2	3	72	2	BB	4,04567441	99,6%	99,6%	0,0%	0	10,077	0
BPAP	t3_BB2	3	72	2	BB	0,07358117	100,0%	100,0%	0,0%	0	10,077	0
BPM	t3_BB2	3	72	2	BB	0,0669968	100,0%	100,0%	0,0%	0	10,077	0
BPP	t3_BB2	3	72	2	BB	0,48985827	100,0%	100,0%	0,0%	0	10,077	0
BPBP	t3_BB2	3	72	2	BB	0,08178592	100,0%	100,0%	0,0%	0	10,077	0
BPPH	t3_BB2	3	72	2	BB	0,05098646	100,0%	100,0%	0,0%	0	10,077	0
BPFL	t3_BB2	3	72	2	BB	0,07890223	100,0%	100,0%	0,0%	0	10,077	0
22BPF	t3_BB3	3	72	3	BB	0,68079217	99,9%	99,9%	0,0%	0	11,932	0
BPAF	t3_BB3	3	72	3	BB	0,4123404	100,0%	100,0%	0,0%	0	11,932	0
24BPF	t3_BB3	3	72	3	BB	1,11460323	99,9%	99,9%	0,0%	0	11,932	0
44BPF	t3_BB3	3	72	3	BB	1,11276928	99,9%	99,9%	0,0%	0	11,932	0
BPE	t3_BB3	3	72	3	BB	0,26633738	100,0%	100,0%	0,0%	0	11,932	0
BPA	t3_BB3	3	72	3	BB	0	100,0%	100,0%	0,0%	0	11,932	0
BPC	t3_BB3	3	72	3	BB	0,16454667	100,0%	100,0%	0,0%	0	11,932	0
BPB	t3_BB3	3	72	3	BB	0,04294423	100,0%	100,0%	0,0%	0	11,932	0
BP26DM	t3_BB3	3	72	3	BB	0,0733854	100,0%	100,0%	0,0%	0	11,932	0
BPC2	t3_BB3	3	72	3	BB	0,14828269	100,0%	100,0%	0,0%	0	11,932	0
BPZ	t3_BB3	3	72	3	BB	0,14721978	100,0%	100,0%	0,0%	0	11,932	0
BPS	t3_BB3	3	72	3	BB	2,95026178	99,7%	99,7%	0,0%	0	11,932	0
BPAP	t3_BB3	3	72	3	BB	0,2888648	100,0%	100,0%	0,0%	0	11,932	0
BPM	t3_BB3	3	72	3	BB	0,02998668	100,0%	100,0%	0,0%	0	11,932	0
BPP	t3_BB3	3	72	3	BB	0,64703412	99,9%	99,9%	0,0%	0	11,932	0
BPBP	t3_BB3	3	72	3	BB	0,12640079	100,0%	100,0%	0,0%	0	11,932	0
BPPH	t3_BB3	3	72	3	BB	0,13185722	100,0%	100,0%	0,0%	0	11,932	0
BPFL	t3_BB3	3	72	3	BB	0,1542849	100,0%	100,0%	0,0%	0	11,932	0
22BPF	t3_BK1	3	72	1	BK	0,18478498	100,0%	100,0%	0,0%	2,826	2,571	1,099183
BPAF	t3_BK1	3	72	1	BK	0,63042816	99,9%	99,9%	0,0%	2,826	2,571	1,099183
24BPF	t3_BK1	3	72	1	BK	0,8164058	99,9%	99,9%	0,0%	2,826	2,571	1,099183
44BPF	t3_BK1	3	72	1	BK	1,00715591	99,9%	99,9%	0,0%	2,826	2,571	1,099183
BPE	t3_BK1	3	72	1	BK	0,46232739	100,0%	100,0%	0,0%	2,826	2,571	1,099183
BPA	t3_BK1	3	72	1	BK	0	100,0%	100,0%	0,0%	2,826	2,571	1,099183
BPC	t3_BK1	3	72	1	BK	0,05625042	100,0%	100,0%	0,0%	2,826	2,571	1,099183
BPB	t3_BK1	3	72	1	BK	0,18000903	100,0%	100,0%	0,0%	2,826	2,571	1,099183
BP26DM	t3_BK1	3	72	1	BK	0,21224634	100,0%	100,0%	0,0%	2,826	2,571	1,099183
BPC2	t3_BK1	3	72	1	BK	0,22761263	100,0%	100,0%	0,0%	2,826	2,571	1,099183
BPZ	t3_BK1	3	72	1	BK	0,14598076	100,0%	100,0%	0,0%	2,826	2,571	1,099183
BPS	t3_BK1	3	72	1	BK	9,11640715	99,1%	99,1%	0,0%	2,826	2,571	1,099183
BPAP	t3_BK1	3	72	1	BK	0,24603842	100,0%	100,0%	0,0%	2,826	2,571	1,099183
BPM	t3_BK1	3	72	1	BK	0,28699193	100,0%	100,0%	0,0%	2,826	2,571	1,099183
BPP	t3_BK1	3	72	1	BK	0,75300528	99,9%	99,9%	0,0%	2,826	2,571	1,099183
BPBP	t3_BK1	3	72	1	BK	0,40928471	100,0%	100,0%	0,0%	2,826	2,571	1,099183
BPPH	t3_BK1	3	72	1	BK	0,1976808	100,0%	100,0%	0,0%	2,826	2,571	1,099183
BPFL	t3_BK1	3	72	1	BK	0,78968323	99,9%	99,9%	0,0%	2,826	2,571	1,099183
22BPF	t3_BK2	3	72	2	BK	0,39033059	100,0%	100,0%	0,0%	2,494	2,983	0,836071
BPAF	t3_BK2	3	72	2	BK	0,90859579	99,9%	99,9%	0,0%	2,494	2,983	0,836071
24BPF	t3_BK2	3	72	2	BK	0,56321411	99,9%	99,9%	0,0%	2,494	2,983	0,836071
44BPF	t3_BK2	3	72	2	BK	0,833263	99,9%	99,9%	0,0%	2,494	2,983	0,836071
BPE	t3_BK2	3	72	2	BK	0,37469676	100,0%	100,0%	0,0%	2,494	2,983	0,836071
BPA	t3_BK2	3	72	2	BK	0	100,0%	100,0%	0,0%	2,494	2,983	0,836071
BPC	t3_BK2	3	72	2	BK	0,05827032	100,0%	100,0%	0,0%	2,494	2,983	0,836071
BPB	t3_BK2	3	72	2	BK	0,23731168	100,0%	100,0%	0,0%	2,494	2,983	0,836071
BP26DM	t3_BK2	3	72	2	BK	2,56047783	99,7%	99,7%	0,0%	2,494	2,983	0,836071
BPC2	t3_BK2	3	72	2	BK	0,48646526	100,0%	100,0%	0,0%	2,494	2,983	0,836071
BPZ	t3_BK2	3	72	2	BK	0,28431943	100,0%	100,0%	0,0%	2,494	2,983	0,836071
BPS	t3_BK2	3	72	2	BK	4,98292413	99,5%	99,5%	0,0%	2,494	2,983	0,836071
BPAP	t3_BK2	3	72	2	BK	0,62056754	99,9%	99,9%	0,0%	2,494	2,983	0,836071
BPM	t3_BK2	3	72	2	BK	0,01952841	100,0%	100,0%	0,0%	2,494	2,983	0,836071
BPP	t3_BK2	3	72	2	BK	0,74301916	99,9%	99,9%	0,0%	2,494	2,983	0,836071
BPBP	t3_BK2	3	72	2	BK	0,36686822	100,0%	100,0%	0,0%	2,494	2,983	0,836071
BPPH	t3_BK2	3	72	2	BK	0,07215003	100,0%	100,0%	0,0%	2,494	2,983	0,836071
BPFL	t3_BK2	3	72	2	BK	0,20709695	100,0%	100,0%	0,0%	2,494	2,983	0,836071
22BPF	t3_BK3	3	72	3	BK	0,99640941	99,9%	99,9%	0,0%	2,273	2,894	0,785418
BPAF	t3_BK3	3	72	3	BK	0,74764507	99,9%	99,9%	0,0%	2,273	2,894	0,785418

24BPF	t3_BK3	3	72	3	BK	0,84402937	99,9%	99,9%	0,0%	2,273	2,894	0,785418
44BPF	t3_BK3	3	72	3	BK	0,44532534	100,0%	100,0%	0,0%	2,273	2,894	0,785418
BPE	t3_BK3	3	72	3	BK	0,39797601	100,0%	100,0%	0,0%	2,273	2,894	0,785418
BPA	t3_BK3	3	72	3	BK	0	100,0%	100,0%	0,0%	2,273	2,894	0,785418
BPC	t3_BK3	3	72	3	BK	0,17410489	100,0%	100,0%	0,0%	2,273	2,894	0,785418
BPB	t3_BK3	3	72	3	BK	0,34551312	100,0%	100,0%	0,0%	2,273	2,894	0,785418
BP26DM	t3_BK3	3	72	3	BK	0,27118021	100,0%	100,0%	0,0%	2,273	2,894	0,785418
BPC2	t3_BK3	3	72	3	BK	0,2	100,0%	100,0%	0,0%	2,273	2,894	0,785418
BPZ	t3_BK3	3	72	3	BK	0,36147025	100,0%	100,0%	0,0%	2,273	2,894	0,785418
BPS	t3_BK3	3	72	3	BK	3,36223378	99,7%	99,7%	0,0%	2,273	2,894	0,785418
BPAP	t3_BK3	3	72	3	BK	0,32702747	100,0%	100,0%	0,0%	2,273	2,894	0,785418
BPM	t3_BK3	3	72	3	BK	0,08132636	100,0%	100,0%	0,0%	2,273	2,894	0,785418
BPP	t3_BK3	3	72	3	BK	0,60757407	99,9%	99,9%	0,0%	2,273	2,894	0,785418
BPBP	t3_BK3	3	72	3	BK	0,14649582	100,0%	100,0%	0,0%	2,273	2,894	0,785418
BPPH	t3_BK3	3	72	3	BK	0,14981052	100,0%	100,0%	0,0%	2,273	2,894	0,785418
BPFL	t3_BK3	3	72	3	BK	0,44821795	100,0%	100,0%	0,0%	2,273	2,894	0,785418
22BPF	t3_EA1	3	72	1	EA	917,430344	8,3%	8,3%	0,0%	2,371	0,102	23,2451
BPAF	t3_EA1	3	72	1	EA	872,390918	12,8%	12,8%	0,0%	2,371	0,102	23,2451
24BPF	t3_EA1	3	72	1	EA	763,903122	23,6%	23,6%	0,0%	2,371	0,102	23,2451
44BPF	t3_EA1	3	72	1	EA	791,680344	20,8%	20,8%	0,0%	2,371	0,102	23,2451
BPE	t3_EA1	3	72	1	EA	804,902308	19,5%	19,5%	0,0%	2,371	0,102	23,2451
BPA	t3_EA1	3	72	1	EA	807,990659	19,2%	19,2%	0,0%	2,371	0,102	23,2451
BPC	t3_EA1	3	72	1	EA	750,443697	25,0%	25,0%	0,0%	2,371	0,102	23,2451
BPB	t3_EA1	3	72	1	EA	769,549394	23,0%	23,0%	0,0%	2,371	0,102	23,2451
BP26DM	t3_EA1	3	72	1	EA	668,642814	33,1%	33,1%	0,0%	2,371	0,102	23,2451
BPC2	t3_EA1	3	72	1	EA	271,936024	72,8%	72,8%	0,0%	2,371	0,102	23,2451
BPZ	t3_EA1	3	72	1	EA	742,949901	25,7%	25,7%	0,0%	2,371	0,102	23,2451
BPS	t3_EA1	3	72	1	EA	728,247618	27,2%	27,2%	0,0%	2,371	0,102	23,2451
BPAP	t3_EA1	3	72	1	EA	856,800897	14,3%	14,3%	0,0%	2,371	0,102	23,2451
BPM	t3_EA1	3	72	1	EA	336,173931	66,4%	66,4%	0,0%	2,371	0,102	23,2451
BPP	t3_EA1	3	72	1	EA	207,049254	79,3%	79,3%	0,0%	2,371	0,102	23,2451
BPBP	t3_EA1	3	72	1	EA	428,651715	57,1%	57,1%	0,0%	2,371	0,102	23,2451
BPPH	t3_EA1	3	72	1	EA	41,642861	95,8%	95,8%	0,0%	2,371	0,102	23,2451
BPFL	t3_EA1	3	72	1	EA	420,83236	57,9%	57,9%	0,0%	2,371	0,102	23,2451
22BPF	t3_EA2	3	72	2	EA	853,360333	14,7%	14,7%	0,0%	1,563	0,223	7,008969
BPAF	t3_EA2	3	72	2	EA	901,040658	9,9%	9,9%	0,0%	1,563	0,223	7,008969
24BPF	t3_EA2	3	72	2	EA	934,213907	6,6%	6,6%	0,0%	1,563	0,223	7,008969
44BPF	t3_EA2	3	72	2	EA	729,811613	27,0%	27,0%	0,0%	1,563	0,223	7,008969
BPE	t3_EA2	3	72	2	EA	766,564172	23,3%	23,3%	0,0%	1,563	0,223	7,008969
BPA	t3_EA2	3	72	2	EA	718,38545	28,2%	28,2%	0,0%	1,563	0,223	7,008969
BPC	t3_EA2	3	72	2	EA	682,508862	31,7%	31,7%	0,0%	1,563	0,223	7,008969
BPB	t3_EA2	3	72	2	EA	739,944757	26,0%	26,0%	0,0%	1,563	0,223	7,008969
BP26DM	t3_EA2	3	72	2	EA	533,90568	46,6%	46,6%	0,0%	1,563	0,223	7,008969
BPC2	t3_EA2	3	72	2	EA	46,1733775	95,4%	95,4%	0,0%	1,563	0,223	7,008969
BPZ	t3_EA2	3	72	2	EA	687,897809	31,2%	31,2%	0,0%	1,563	0,223	7,008969
BPS	t3_EA2	3	72	2	EA	707,749439	29,2%	29,2%	0,0%	1,563	0,223	7,008969
BPAP	t3_EA2	3	72	2	EA	666,333007	33,4%	33,4%	0,0%	1,563	0,223	7,008969
BPM	t3_EA2	3	72	2	EA	479,633616	52,0%	52,0%	0,0%	1,563	0,223	7,008969
BPP	t3_EA2	3	72	2	EA	369,461846	63,1%	63,1%	0,0%	1,563	0,223	7,008969
BPBP	t3_EA2	3	72	2	EA	645,978089	35,4%	35,4%	0,0%	1,563	0,223	7,008969
BPPH	t3_EA2	3	72	2	EA	77,3075063	92,3%	92,3%	0,0%	1,563	0,223	7,008969
BPFL	t3_EA2	3	72	2	EA	639,494833	36,1%	36,1%	0,0%	1,563	0,223	7,008969
22BPF	t3_EA3	3	72	3	EA	872,450896	12,8%	12,8%	0,0%	1,631	0,295	5,528814
BPAF	t3_EA3	3	72	3	EA	811,859942	18,8%	18,8%	0,0%	1,631	0,295	5,528814
24BPF	t3_EA3	3	72	3	EA	832,373708	16,8%	16,8%	0,0%	1,631	0,295	5,528814
44BPF	t3_EA3	3	72	3	EA	836,314846	16,4%	16,4%	0,0%	1,631	0,295	5,528814
BPE	t3_EA3	3	72	3	EA	833,840827	16,6%	16,6%	0,0%	1,631	0,295	5,528814
BPA	t3_EA3	3	72	3	EA	816,170572	18,4%	18,4%	0,0%	1,631	0,295	5,528814
BPC	t3_EA3	3	72	3	EA	759,841597	24,0%	24,0%	0,0%	1,631	0,295	5,528814
BPB	t3_EA3	3	72	3	EA	831,28958	16,9%	16,9%	0,0%	1,631	0,295	5,528814
BP26DM	t3_EA3	3	72	3	EA	601,224896	39,9%	39,9%	0,0%	1,631	0,295	5,528814
BPC2	t3_EA3	3	72	3	EA	26,2056745	97,4%	97,4%	0,0%	1,631	0,295	5,528814
BPZ	t3_EA3	3	72	3	EA	784,475987	21,6%	21,6%	0,0%	1,631	0,295	5,528814
BPS	t3_EA3	3	72	3	EA	776,68558	22,3%	22,3%	0,0%	1,631	0,295	5,528814
BPAP	t3_EA3	3	72	3	EA	829,596988	17,0%	17,0%	0,0%	1,631	0,295	5,528814
BPM	t3_EA3	3	72	3	EA	491,845646	50,8%	50,8%	0,0%	1,631	0,295	5,528814
BPP	t3_EA3	3	72	3	EA	350,109343	65,0%	65,0%	0,0%	1,631	0,295	5,528814
BPBP	t3_EA3	3	72	3	EA	570,867435	42,9%	42,9%	0,0%	1,631	0,295	5,528814
BPPH	t3_EA3	3	72	3	EA	63,0843245	93,7%	93,7%	0,0%	1,631	0,295	5,528814
BPFL	t3_EA3	3	72	3	EA	588,612281	41,1%	41,1%	0,0%	1,631	0,295	5,528814
22BPF	t3_EB1	3	72	1	EB	822,034437	17,8%	17,8%	0,0%	0	10,592	0
BPAF	t3_EB1	3	72	1	EB	717,768498	28,2%	28,2%	0,0%	0	10,592	0
24BPF	t3_EB1	3	72	1	EB	767,933325	23,2%	23,2%	0,0%	0	10,592	0
44BPF	t3_EB1	3	72	1	EB	817,061631	18,3%	18,3%	0,0%	0	10,592	0
BPE	t3_EB1	3	72	1	EB	768,574225	23,1%	23,1%	0,0%	0	10,592	0
BPA	t3_EB1	3	72	1	EB	679,459581	32,1%	32,1%	0,0%	0	10,592	0
BPC	t3_EB1	3	72	1	EB	752,481665	24,8%	24,8%	0,0%	0	10,592	0
BPB	t3_EB1	3	72	1	EB	747,972661	25,2%	25,2%	0,0%	0	10,592	0
BP26DM	t3_EB1	3	72	1	EB	706,581946	29,3%	29,3%	0,0%	0	10,592	0
BPC2	t3_EB1	3	72	1	EB	626,313684	37,4%	37,4%	0,0%	0	10,592	0
BPZ	t3_EB1	3	72	1	EB	704,674357	29,5%	29,5%	0,0%	0	10,592	0
BPS	t3_EB1	3	72	1	EB	784,992146	21,5%	21,5%	0,0%	0	10,592	0
BPAP	t3_EB1	3	72	1	EB	664,765744	33,5%	33,5%	0,0%	0	10,592	0
BPM	t3_EB1	3	72	1	EB	243,212645	75,7%	75,7%	0,0%	0	10,592	0
BPP	t3_EB1	3	72	1	EB	145,405157	85,5%	85,5%	0,0%	0	10,592	0
BPBP	t3_EB1	3	72	1	EB	376,892906	62,3%	62,3%	0,0%	0	10,592	0

BPPH	t3_EB1	3	72	1	EB	61,4135754	93,9%	93,9%	0,0%	0	10,592	0
BPFL	t3_EB1	3	72	1	EB	435,326293	56,5%	56,5%	0,0%	0	10,592	0
22BPF	t3_EB2	3	72	2	EB	919,797753	8,0%	8,0%	0,0%	0	9,905	0
BPAF	t3_EB2	3	72	2	EB	792,718214	20,7%	20,7%	0,0%	0	9,905	0
24BPF	t3_EB2	3	72	2	EB	873,948784	12,6%	12,6%	0,0%	0	9,905	0
44BPF	t3_EB2	3	72	2	EB	776,292698	22,4%	22,4%	0,0%	0	9,905	0
BPE	t3_EB2	3	72	2	EB	723,213697	27,7%	27,7%	0,0%	0	9,905	0
BPA	t3_EB2	3	72	2	EB	696,948275	30,3%	30,3%	0,0%	0	9,905	0
BPC	t3_EB2	3	72	2	EB	781,942532	21,8%	21,8%	0,0%	0	9,905	0
BPB	t3_EB2	3	72	2	EB	766,551807	23,3%	23,3%	0,0%	0	9,905	0
BP26DM	t3_EB2	3	72	2	EB	780,058712	22,0%	22,0%	0,0%	0	9,905	0
BPC2	t3_EB2	3	72	2	EB	684,709551	31,5%	31,5%	0,0%	0	9,905	0
BPZ	t3_EB2	3	72	2	EB	755,478714	24,5%	24,5%	0,0%	0	9,905	0
BPS	t3_EB2	3	72	2	EB	786,438591	21,4%	21,4%	0,0%	0	9,905	0
BPAP	t3_EB2	3	72	2	EB	747,482893	25,3%	25,3%	0,0%	0	9,905	0
BPM	t3_EB2	3	72	2	EB	274,937731	72,5%	72,5%	0,0%	0	9,905	0
BPP	t3_EB2	3	72	2	EB	172,226563	82,8%	82,8%	0,0%	0	9,905	0
BPBP	t3_EB2	3	72	2	EB	408,364035	59,2%	59,2%	0,0%	0	9,905	0
BPPH	t3_EB2	3	72	2	EB	82,1403048	91,8%	91,8%	0,0%	0	9,905	0
BPFL	t3_EB2	3	72	2	EB	497,534413	50,2%	50,2%	0,0%	0	9,905	0
22BPF	t3_EB3	3	72	3	EB	728,297697	27,2%	27,2%	0,0%	0	10,454	0
BPAF	t3_EB3	3	72	3	EB	646,471307	35,4%	35,4%	0,0%	0	10,454	0
24BPF	t3_EB3	3	72	3	EB	696,976363	30,3%	30,3%	0,0%	0	10,454	0
44BPF	t3_EB3	3	72	3	EB	757,630182	24,2%	24,2%	0,0%	0	10,454	0
BPE	t3_EB3	3	72	3	EB	626,261482	37,4%	37,4%	0,0%	0	10,454	0
BPA	t3_EB3	3	72	3	EB	681,808131	31,8%	31,8%	0,0%	0	10,454	0
BPC	t3_EB3	3	72	3	EB	707,644063	29,2%	29,2%	0,0%	0	10,454	0
BPB	t3_EB3	3	72	3	EB	706,293356	29,4%	29,4%	0,0%	0	10,454	0
BP26DM	t3_EB3	3	72	3	EB	688,595097	31,1%	31,1%	0,0%	0	10,454	0
BPC2	t3_EB3	3	72	3	EB	608,044327	39,2%	39,2%	0,0%	0	10,454	0
BPZ	t3_EB3	3	72	3	EB	687,362994	31,3%	31,3%	0,0%	0	10,454	0
BPS	t3_EB3	3	72	3	EB	758,953125	24,1%	24,1%	0,0%	0	10,454	0
BPAP	t3_EB3	3	72	3	EB	661,204511	33,9%	33,9%	0,0%	0	10,454	0
BPM	t3_EB3	3	72	3	EB	229,566203	77,0%	77,0%	0,0%	0	10,454	0
BPP	t3_EB3	3	72	3	EB	139,759335	86,0%	86,0%	0,0%	0	10,454	0
BPBP	t3_EB3	3	72	3	EB	346,843483	65,3%	65,3%	0,0%	0	10,454	0
BPPH	t3_EB3	3	72	3	EB	62,9458649	93,7%	93,7%	0,0%	0	10,454	0
BPFL	t3_EB3	3	72	3	EB	409,814547	59,0%	59,0%	0,0%	0	10,454	0
22BPF	t3_EK1	3	72	1	EK	723,159149	27,7%	27,7%	0,0%	3,236	3,557	0,909755
BPAF	t3_EK1	3	72	1	EK	725,416821	27,5%	27,5%	0,0%	3,236	3,557	0,909755
24BPF	t3_EK1	3	72	1	EK	677,754366	32,2%	32,2%	0,0%	3,236	3,557	0,909755
44BPF	t3_EK1	3	72	1	EK	720,027959	28,0%	28,0%	0,0%	3,236	3,557	0,909755
BPE	t3_EK1	3	72	1	EK	720,560403	27,9%	27,9%	0,0%	3,236	3,557	0,909755
BPA	t3_EK1	3	72	1	EK	700,941465	29,9%	29,9%	0,0%	3,236	3,557	0,909755
BPC	t3_EK1	3	72	1	EK	609,631633	39,0%	39,0%	0,0%	3,236	3,557	0,909755
BPB	t3_EK1	3	72	1	EK	716,047984	28,4%	28,4%	0,0%	3,236	3,557	0,909755
BP26DM	t3_EK1	3	72	1	EK	395,27028	60,5%	60,5%	0,0%	3,236	3,557	0,909755
BPC2	t3_EK1	3	72	1	EK	25,159482	97,5%	97,5%	0,0%	3,236	3,557	0,909755
BPZ	t3_EK1	3	72	1	EK	678,713064	32,1%	32,1%	0,0%	3,236	3,557	0,909755
BPS	t3_EK1	3	72	1	EK	708,00846	29,2%	29,2%	0,0%	3,236	3,557	0,909755
BPAP	t3_EK1	3	72	1	EK	702,814273	29,7%	29,7%	0,0%	3,236	3,557	0,909755
BPM	t3_EK1	3	72	1	EK	377,926615	62,2%	62,2%	0,0%	3,236	3,557	0,909755
BPP	t3_EK1	3	72	1	EK	274,435032	72,6%	72,6%	0,0%	3,236	3,557	0,909755
BPBP	t3_EK1	3	72	1	EK	486,507935	51,3%	51,3%	0,0%	3,236	3,557	0,909755
BPPH	t3_EK1	3	72	1	EK	54,7032146	94,5%	94,5%	0,0%	3,236	3,557	0,909755
BPFL	t3_EK1	3	72	1	EK	492,343417	50,8%	50,8%	0,0%	3,236	3,557	0,909755
22BPF	t3_EK2	3	72	2	EK	792,821184	20,7%	20,7%	0,0%	2,492	4,236	0,588291
BPAF	t3_EK2	3	72	2	EK	802,152107	19,8%	19,8%	0,0%	2,492	4,236	0,588291
24BPF	t3_EK2	3	72	2	EK	746,428722	25,4%	25,4%	0,0%	2,492	4,236	0,588291
44BPF	t3_EK2	3	72	2	EK	765,05423	23,5%	23,5%	0,0%	2,492	4,236	0,588291
BPE	t3_EK2	3	72	2	EK	752,591383	24,7%	24,7%	0,0%	2,492	4,236	0,588291
BPA	t3_EK2	3	72	2	EK	948,546312	5,1%	5,1%	0,0%	2,492	4,236	0,588291
BPC	t3_EK2	3	72	2	EK	688,654534	31,1%	31,1%	0,0%	2,492	4,236	0,588291
BPB	t3_EK2	3	72	2	EK	799,995266	20,0%	20,0%	0,0%	2,492	4,236	0,588291
BP26DM	t3_EK2	3	72	2	EK	400,043679	60,0%	60,0%	0,0%	2,492	4,236	0,588291
BPC2	t3_EK2	3	72	2	EK	67,7579384	93,2%	93,2%	0,0%	2,492	4,236	0,588291
BPZ	t3_EK2	3	72	2	EK	760,832893	23,9%	23,9%	0,0%	2,492	4,236	0,588291
BPS	t3_EK2	3	72	2	EK	806,45868	19,4%	19,4%	0,0%	2,492	4,236	0,588291
BPAP	t3_EK2	3	72	2	EK	788,028886	21,2%	21,2%	0,0%	2,492	4,236	0,588291
BPM	t3_EK2	3	72	2	EK	387,413073	61,3%	61,3%	0,0%	2,492	4,236	0,588291
BPP	t3_EK2	3	72	2	EK	293,018494	70,7%	70,7%	0,0%	2,492	4,236	0,588291
BPBP	t3_EK2	3	72	2	EK	524,866188	47,5%	47,5%	0,0%	2,492	4,236	0,588291
BPPH	t3_EK2	3	72	2	EK	57,3170789	94,3%	94,3%	0,0%	2,492	4,236	0,588291
BPFL	t3_EK2	3	72	2	EK	573,844541	42,6%	42,6%	0,0%	2,492	4,236	0,588291
22BPF	t3_EK3	3	72	3	EK	818,630283	18,1%	18,1%	0,0%	2,73	2,888	0,945291
BPAF	t3_EK3	3	72	3	EK	797,414524	20,3%	20,3%	0,0%	2,73	2,888	0,945291
24BPF	t3_EK3	3	72	3	EK	772,638044	22,7%	22,7%	0,0%	2,73	2,888	0,945291
44BPF	t3_EK3	3	72	3	EK	778,27137	22,2%	22,2%	0,0%	2,73	2,888	0,945291
BPE	t3_EK3	3	72	3	EK	787,331651	21,3%	21,3%	0,0%	2,73	2,888	0,945291
BPA	t3_EK3	3	72	3	EK	775,815308	22,4%	22,4%	0,0%	2,73	2,888	0,945291
BPC	t3_EK3	3	72	3	EK	641,519242	35,8%	35,8%	0,0%	2,73	2,888	0,945291
BPB	t3_EK3	3	72	3	EK	758,263129	24,2%	24,2%	0,0%	2,73	2,888	0,945291
BP26DM	t3_EK3	3	72	3	EK	363,230042	63,7%	63,7%	0,0%	2,73	2,888	0,945291
BPC2	t3_EK3	3	72	3	EK	70,1399692	93,0%	93,0%	0,0%	2,73	2,888	0,945291
BPZ	t3_EK3	3	72	3	EK	686,596413	31,3%	31,3%	0,0%	2,73	2,888	0,945291
BPS	t3_EK3	3	72	3	EK	790,243754	21,0%	21,0%	0,0%	2,73	2,888	0,945291

BPAP	t3_EK3	3	72	3	EK	706,170035		29,4%	29,4%	0,0%	2,73	2,888	0,945291
BPM	t3_EK3	3	72	3	EK	347,427922		65,3%	65,3%	0,0%	2,73	2,888	0,945291
BPP	t3_EK3	3	72	3	EK	258,290923		74,2%	74,2%	0,0%	2,73	2,888	0,945291
BPBP	t3_EK3	3	72	3	EK	494,539416		50,5%	50,5%	0,0%	2,73	2,888	0,945291
BPPH	t3_EK3	3	72	3	EK	47,4413482		95,3%	95,3%	0,0%	2,73	2,888	0,945291
BPFL	t3_EK3	3	72	3	EK	523,828406		47,6%	47,6%	0,0%	2,73	2,888	0,945291
22BPF	t7_ABS1	7	168	1	ABS	834,324981	0,512223	16,6%	16,5%	-0,1%	0	0,01832	0
24BPF	t7_ABS1	7	168	1	ABS	786,623863	0,522944	21,3%	21,3%	-0,1%	0	0,01832	0
44BPF	t7_ABS1	7	168	1	ABS	864,461535	0,749699	13,6%	13,5%	-0,1%	0	0,01832	0
BP26DM	t7_ABS1	7	168	1	ABS	580,383857	4,816341	42,0%	41,5%	-0,5%	0	0,01832	0
BPA	t7_ABS1	7	168	1	ABS	817,822894	0	18,2%	18,2%	0,0%	0	0,01832	0
BPAF	t7_ABS1	7	168	1	ABS	739,203566	33,86559	26,1%	22,7%	-3,4%	0	0,01832	0
BPAP	t7_ABS1	7	168	1	ABS	816,867794	18,38491	18,3%	16,5%	-1,8%	0	0,01832	0
BPB	t7_ABS1	7	168	1	ABS	855,904598	4,848038	14,4%	13,9%	-0,5%	0	0,01832	0
BPBP	t7_ABS1	7	168	1	ABS	473,062447	191,7661	52,7%	33,5%	-19,2%	0	0,01832	0
BPC	t7_ABS1	7	168	1	ABS	746,934693	5,679425	25,3%	24,7%	-0,6%	0	0,01832	0
BPC2	t7_ABS1	7	168	1	ABS	702,291707	16,50892	29,8%	28,1%	-1,7%	0	0,01832	0
BPE	t7_ABS1	7	168	1	ABS	795,04082	0,938022	20,5%	20,4%	-0,1%	0	0,01832	0
BPFL	t7_ABS1	7	168	1	ABS	403,80521	131,9766	59,6%	46,4%	-13,2%	0	0,01832	0
BPM	t7_ABS1	7	168	1	ABS	424,745031	158,934	57,5%	41,6%	-15,9%	0	0,01832	0
BPP	t7_ABS1	7	168	1	ABS	301,034611	197,1976	69,9%	50,2%	-19,7%	0	0,01832	0
BPPH	t7_ABS1	7	168	1	ABS	168,739529	110,4487	83,1%	72,1%	-11,0%	0	0,01832	0
BPS	t7_ABS1	7	168	1	ABS	803,322077	0,059203	19,7%	19,7%	0,0%	0	0,01832	0
BPZ	t7_ABS1	7	168	1	ABS	793,095054	16,69381	20,7%	19,0%	-1,7%	0	0,01832	0
22BPF	t7_ABS2	7	168	2	ABS	868,104205	0,863875	13,2%	13,1%	-0,1%	0	0,04852	0
24BPF	t7_ABS2	7	168	2	ABS	827,711161	0,739048	17,2%	17,2%	-0,1%	0	0,04852	0
44BPF	t7_ABS2	7	168	2	ABS	828,941212	0,417532	17,1%	17,1%	0,0%	0	0,04852	0
BP26DM	t7_ABS2	7	168	2	ABS	646,071645	8,579217	35,4%	34,5%	-0,9%	0	0,04852	0
BPA	t7_ABS2	7	168	2	ABS	762,766474	0	23,7%	23,7%	0,0%	0	0,04852	0
BPAF	t7_ABS2	7	168	2	ABS	829,815278	46,11661	17,0%	12,4%	-4,6%	0	0,04852	0
BPAP	t7_ABS2	7	168	2	ABS	818,477218	20,44844	18,2%	16,1%	-2,0%	0	0,04852	0
BPB	t7_ABS2	7	168	2	ABS	811,687659	4,373567	18,8%	18,4%	-0,4%	0	0,04852	0
BPBP	t7_ABS2	7	168	2	ABS	411,12426	195,9609	58,9%	39,3%	-19,6%	0	0,04852	0
BPC	t7_ABS2	7	168	2	ABS	752,491795	6,792995	24,8%	24,1%	-0,7%	0	0,04852	0
BPC2	t7_ABS2	7	168	2	ABS	702,496451	18,36369	29,8%	27,9%	-1,8%	0	0,04852	0
BPE	t7_ABS2	7	168	2	ABS	865,316411	1,084666	13,5%	13,4%	-0,1%	0	0,04852	0
BPFL	t7_ABS2	7	168	2	ABS	365,072685	132,148	63,5%	50,3%	-13,2%	0	0,04852	0
BPM	t7_ABS2	7	168	2	ABS	398,041291	183,4079	60,2%	41,9%	-18,3%	0	0,04852	0
BPP	t7_ABS2	7	168	2	ABS	242,625601	231,2294	75,7%	52,6%	-23,1%	0	0,04852	0
BPPH	t7_ABS2	7	168	2	ABS	115,508963	150,2047	88,4%	73,4%	-15,0%	0	0,04852	0
BPS	t7_ABS2	7	168	2	ABS	764,401722	0,079532	23,6%	23,6%	0,0%	0	0,04852	0
BPZ	t7_ABS2	7	168	2	ABS	816,980917	17,62469	18,3%	16,5%	-1,8%	0	0,04852	0
22BPF	t7_ABS3	7	168	3	ABS	825,593097	0,666095	17,4%	17,4%	-0,1%	0	0,01446	0
24BPF	t7_ABS3	7	168	3	ABS	814,067375	0,56525	18,6%	18,5%	-0,1%	0	0,01446	0
44BPF	t7_ABS3	7	168	3	ABS	838,753784	0,813807	16,1%	16,0%	-0,1%	0	0,01446	0
BP26DM	t7_ABS3	7	168	3	ABS	709,031233	5,964941	29,1%	28,5%	-0,6%	0	0,01446	0
BPA	t7_ABS3	7	168	3	ABS	764,246943	0	23,6%	23,6%	0,0%	0	0,01446	0
BPAF	t7_ABS3	7	168	3	ABS	720,510692	29,03391	27,9%	25,0%	-2,9%	0	0,01446	0
BPAP	t7_ABS3	7	168	3	ABS	779,542406	17,49054	22,0%	20,3%	-1,7%	0	0,01446	0
BPB	t7_ABS3	7	168	3	ABS	792,396354	3,810581	20,8%	20,4%	-0,4%	0	0,01446	0
BPBP	t7_ABS3	7	168	3	ABS	418,516445	163,9123	58,1%	41,8%	-16,4%	0	0,01446	0
BPC	t7_ABS3	7	168	3	ABS	751,028023	5,060162	24,9%	24,4%	-0,5%	0	0,01446	0
BPC2	t7_ABS3	7	168	3	ABS	691,063956	14,58574	30,9%	29,4%	-1,5%	0	0,01446	0
BPE	t7_ABS3	7	168	3	ABS	812,532884	0,730071	18,7%	18,7%	-0,1%	0	0,01446	0
BPFL	t7_ABS3	7	168	3	ABS	388,581952	118,331	61,1%	49,3%	-11,8%	0	0,01446	0
BPM	t7_ABS3	7	168	3	ABS	430,467192	134,2845	57,0%	43,5%	-13,4%	0	0,01446	0
BPP	t7_ABS3	7	168	3	ABS	301,056501	171,6231	69,9%	52,7%	-17,2%	0	0,01446	0
BPPH	t7_ABS3	7	168	3	ABS	190,436514	100,035	81,0%	71,0%	-10,0%	0	0,01446	0
BPS	t7_ABS3	7	168	3	ABS	786,100984	0,097157	21,4%	21,4%	0,0%	0	0,01446	0
BPZ	t7_ABS3	7	168	3	ABS	783,225114	14,67922	21,7%	20,2%	-1,5%	0	0,01446	0
22BPF	t7_ABT1	7	168	1	ABT	867,698835	0,2	13,2%	13,2%	0,0%	0	0,12303	0
24BPF	t7_ABT1	7	168	1	ABT	880,350217	0,430006	12,0%	11,9%	0,0%	0	0,12303	0
44BPF	t7_ABT1	7	168	1	ABT	892,448958	0,549905	10,8%	10,7%	-0,1%	0	0,12303	0
BP26DM	t7_ABT1	7	168	1	ABT	670,65954	5,646385	32,9%	32,4%	-0,6%	0	0,12303	0
BPA	t7_ABT1	7	168	1	ABT	856,520875	0	14,3%	14,3%	0,0%	0	0,12303	0
BPAF	t7_ABT1	7	168	1	ABT	768,675687	35,32619	23,1%	19,6%	-3,5%	0	0,12303	0
BPAP	t7_ABT1	7	168	1	ABT	805,906083	22,4027	19,4%	17,2%	-2,2%	0	0,12303	0
BPB	t7_ABT1	7	168	1	ABT	916,522357	5,754659	8,3%	7,8%	-0,6%	0	0,12303	0
BPBP	t7_ABT1	7	168	1	ABT	439,542934	206,7797	56,0%	35,4%	-20,7%	0	0,12303	0
BPC	t7_ABT1	7	168	1	ABT	848,657358	6,387487	15,1%	14,5%	-0,6%	0	0,12303	0
BPC2	t7_ABT1	7	168	1	ABT	821,065143	19,70469	17,9%	15,9%	-2,0%	0	0,12303	0
BPE	t7_ABT1	7	168	1	ABT	864,277879	1,038119	13,6%	13,5%	-0,1%	0	0,12303	0
BPFL	t7_ABT1	7	168	1	ABT	429,673091	143,5552	57,0%	42,7%	-14,4%	0	0,12303	0
BPM	t7_ABT1	7	168	1	ABT	386,511718	159,7116	61,3%	45,4%	-16,0%	0	0,12303	0
BPP	t7_ABT1	7	168	1	ABT	267,292585	197,1077	73,3%	53,6%	-19,7%	0	0,12303	0
BPPH	t7_ABT1	7	168	1	ABT	141,498088	97,22695	85,9%	76,1%	-9,7%	0	0,12303	0
BPS	t7_ABT1	7	168	1	ABT	875,927475	0,164481	12,4%	12,4%	0,0%	0	0,12303	0
BPZ	t7_ABT1	7	168	1	ABT	867,994268	19,99582	13,2%	11,2%	-2,0%	0	0,12303	0
22BPF	t7_ABT2	7	168	2	ABT	847,001091	1,538238	15,3%	15,1%	-0,2%	0,00004	0,08444	0,000474
24BPF	t7_ABT2	7	168	2	ABT	799,749119	1,587612	20,0%	19,9%	-0,2%	0,00004	0,08444	0,000474
44BPF	t7_ABT2	7	168	2	ABT	872,633511	1,622277	12,7%	12,6%	-0,2%	0,00004	0,08444	0,000474
BP26DM	t7_ABT2	7	168	2	ABT	622,849459	11,28563	37,7%	36,6%	-1,1%	0,00004	0,08444	0,000474
BPA	t7_ABT2	7	168	2	ABT	788,347242	2,070302	21,2%	21,0%	-0,2%	0,00004	0,08444	0,000474
BPAF	t7_ABT2	7	168	2	ABT	716,706811	46,63258	28,3%	23,7%	-4,7%	0,00004	0,08444	0,000474
BPAP	t7_ABT2	7	168	2	ABT	821,938942	27,22311	17,8%	15,1%	-2,7%	0,00004	0,08444	0,000474
BPB	t7_ABT2	7	168	2	ABT	872,465718	6,814935	12,8%	12,1%	-0,7%	0,00004	0,08444	0,000474

BPBP	t7_ABT2	7	168	2	ABT	438,103746	257,0321	56,2%	30,5%	-25,7%	0,00004	0,08444	0,000474
BPC	t7_ABT2	7	168	2	ABT	795,671934	9,220539	20,4%	19,5%	-0,9%	0,00004	0,08444	0,000474
BPC2	t7_ABT2	7	168	2	ABT	774,521821	24,55649	22,5%	20,1%	-2,5%	0,00004	0,08444	0,000474
BPE	t7_ABT2	7	168	2	ABT	802,981797	1,563283	19,7%	19,5%	-0,2%	0,00004	0,08444	0,000474
BPFL	t7_ABT2	7	168	2	ABT	416,776069	172,7838	58,3%	41,0%	-17,3%	0,00004	0,08444	0,000474
BPM	t7_ABT2	7	168	2	ABT	337,151316	238,2889	66,3%	42,5%	-23,8%	0,00004	0,08444	0,000474
BPP	t7_ABT2	7	168	2	ABT	245,012974	305,6762	75,5%	44,9%	-30,6%	0,00004	0,08444	0,000474
BPPH	t7_ABT2	7	168	2	ABT	112,773872	220,5157	88,7%	66,7%	-22,1%	0,00004	0,08444	0,000474
BPS	t7_ABT2	7	168	2	ABT	868,803786	0,097862	13,1%	13,1%	0,0%	0,00004	0,08444	0,000474
BPZ	t7_ABT2	7	168	2	ABT	791,232833	24,38166	20,9%	18,4%	-2,4%	0,00004	0,08444	0,000474
22BPF	t7_ABT3	7	168	3	ABT	779,494006	0,946007	22,1%	22,0%	-0,1%	0,00008	0,1724	0,000464
24BPF	t7_ABT3	7	168	3	ABT	805,036847	0,823305	19,5%	19,4%	-0,1%	0,00008	0,1724	0,000464
44BPF	t7_ABT3	7	168	3	ABT	812,051648	0,514881	18,8%	18,7%	-0,1%	0,00008	0,1724	0,000464
BP26DM	t7_ABT3	7	168	3	ABT	485,378022	8,065626	51,5%	50,7%	-0,8%	0,00008	0,1724	0,000464
BPA	t7_ABT3	7	168	3	ABT	860,98625	0	13,9%	13,9%	0,0%	0,00008	0,1724	0,000464
BPAF	t7_ABT3	7	168	3	ABT	648,433467	36,19368	35,2%	31,5%	-3,6%	0,00008	0,1724	0,000464
BPAP	t7_ABT3	7	168	3	ABT	715,070445	23,8911	28,5%	26,1%	-2,4%	0,00008	0,1724	0,000464
BPB	t7_ABT3	7	168	3	ABT	804,097672	5,887014	19,6%	19,0%	-0,6%	0,00008	0,1724	0,000464
BPBP	t7_ABT3	7	168	3	ABT	335,585933	182,6208	66,4%	48,2%	-18,3%	0,00008	0,1724	0,000464
BPC	t7_ABT3	7	168	3	ABT	704,89782	6,617992	29,5%	28,8%	-0,7%	0,00008	0,1724	0,000464
BPC2	t7_ABT3	7	168	3	ABT	676,127216	19,97673	32,4%	30,4%	-2,0%	0,00008	0,1724	0,000464
BPE	t7_ABT3	7	168	3	ABT	779,811757	0,966345	22,0%	21,9%	-0,1%	0,00008	0,1724	0,000464
BPFL	t7_ABT3	7	168	3	ABT	334,013027	129,3476	66,6%	53,7%	-12,9%	0,00008	0,1724	0,000464
BPM	t7_ABT3	7	168	3	ABT	297,623428	156,3595	70,2%	54,6%	-15,6%	0,00008	0,1724	0,000464
BPP	t7_ABT3	7	168	3	ABT	192,017406	211,9852	80,8%	59,6%	-21,2%	0,00008	0,1724	0,000464
BPPH	t7_ABT3	7	168	3	ABT	98,875932	109,2587	90,1%	79,2%	-10,9%	0,00008	0,1724	0,000464
BPS	t7_ABT3	7	168	3	ABT	772,709003	0,107606	22,7%	22,7%	0,0%	0,00008	0,1724	0,000464
BPZ	t7_ABT3	7	168	3	ABT	710,858195	19,87297	28,9%	26,9%	-2,0%	0,00008	0,1724	0,000464
22BPF	t7_BA1	7	168	1	BA	1,17847409	0,184343	99,9%	99,9%	0,0%	2,82435	0,26307	10,73612
24BPF	t7_BA1	7	168	1	BA	0,81425256	0,25742	99,9%	99,9%	0,0%	2,82435	0,26307	10,73612
44BPF	t7_BA1	7	168	1	BA	1,70626308	0,370699	99,8%	99,8%	0,0%	2,82435	0,26307	10,73612
BP26DM	t7_BA1	7	168	1	BA	0,35811407	0,176679	100,0%	99,9%	0,0%	2,82435	0,26307	10,73612
BPA	t7_BA1	7	168	1	BA	0	30,12555	100,0%	97,0%	-3,0%	2,82435	0,26307	10,73612
BPAF	t7_BA1	7	168	1	BA	1,75789531	0,213958	99,8%	99,8%	0,0%	2,82435	0,26307	10,73612
BPAP	t7_BA1	7	168	1	BA	0,92406834	0,246986	99,9%	99,9%	0,0%	2,82435	0,26307	10,73612
BPB	t7_BA1	7	168	1	BA	1,18106858	0,10249	99,9%	99,9%	0,0%	2,82435	0,26307	10,73612
BPBP	t7_BA1	7	168	1	BA	0,8628823	0,057151	99,9%	99,9%	0,0%	2,82435	0,26307	10,73612
BPC	t7_BA1	7	168	1	BA	0,49512943	0,113845	100,0%	99,9%	0,0%	2,82435	0,26307	10,73612
BPC2	t7_BA1	7	168	1	BA	0,19744078	0,201418	100,0%	100,0%	0,0%	2,82435	0,26307	10,73612
BPE	t7_BA1	7	168	1	BA	0,82500893	0,150997	99,9%	99,9%	0,0%	2,82435	0,26307	10,73612
BPFL	t7_BA1	7	168	1	BA	0,13181475	0,473721	100,0%	99,9%	0,0%	2,82435	0,26307	10,73612
BPM	t7_BA1	7	168	1	BA	0,05799968	0,035825	100,0%	100,0%	0,0%	2,82435	0,26307	10,73612
BPP	t7_BA1	7	168	1	BA	1,03868194	0,688354	99,9%	99,8%	-0,1%	2,82435	0,26307	10,73612
BPPH	t7_BA1	7	168	1	BA	0,35032451	0,170829	100,0%	99,9%	0,0%	2,82435	0,26307	10,73612
BPS	t7_BA1	7	168	1	BA	7,48914564	2,681924	99,3%	99,0%	-0,3%	2,82435	0,26307	10,73612
BPZ	t7_BA1	7	168	1	BA	0,65884478	0,243962	99,9%	99,9%	0,0%	2,82435	0,26307	10,73612
22BPF	t7_BA2	7	168	2	BA	0,98953374	0,381125	99,9%	99,9%	0,0%	4,898	0,29461	16,62537
24BPF	t7_BA2	7	168	2	BA	0,6998241	0,450972	99,9%	99,9%	0,0%	4,898	0,29461	16,62537
44BPF	t7_BA2	7	168	2	BA	2,12223778	0,103188	99,8%	99,8%	0,0%	4,898	0,29461	16,62537
BP26DM	t7_BA2	7	168	2	BA	0,27824357	0,07041	100,0%	100,0%	0,0%	4,898	0,29461	16,62537
BPA	t7_BA2	7	168	2	BA	0	26,64008	100,0%	97,3%	-2,7%	4,898	0,29461	16,62537
BPAF	t7_BA2	7	168	2	BA	0,92930074	0,545362	99,9%	99,9%	-0,1%	4,898	0,29461	16,62537
BPAP	t7_BA2	7	168	2	BA	0,45194645	0,074457	100,0%	99,9%	0,0%	4,898	0,29461	16,62537
BPB	t7_BA2	7	168	2	BA	0,34825008	0,096688	100,0%	100,0%	0,0%	4,898	0,29461	16,62537
BPBP	t7_BA2	7	168	2	BA	0,3530194	0,075355	100,0%	100,0%	0,0%	4,898	0,29461	16,62537
BPC	t7_BA2	7	168	2	BA	0,22619506	0,02	100,0%	100,0%	0,0%	4,898	0,29461	16,62537
BPC2	t7_BA2	7	168	2	BA	0,13487245	0,15846	100,0%	100,0%	0,0%	4,898	0,29461	16,62537
BPE	t7_BA2	7	168	2	BA	0,53744956	0,13688	99,9%	99,9%	0,0%	4,898	0,29461	16,62537
BPFL	t7_BA2	7	168	2	BA	0,13400138	1,703176	100,0%	99,8%	-0,2%	4,898	0,29461	16,62537
BPM	t7_BA2	7	168	2	BA	0,02379982	0,02212	100,0%	100,0%	0,0%	4,898	0,29461	16,62537
BPP	t7_BA2	7	168	2	BA	0,79821034	0,702632	99,9%	99,8%	-0,1%	4,898	0,29461	16,62537
BPPH	t7_BA2	7	168	2	BA	0,01816257	0,016835	100,0%	100,0%	0,0%	4,898	0,29461	16,62537
BPS	t7_BA2	7	168	2	BA	3,26162612	5,615156	99,7%	99,1%	-0,6%	4,898	0,29461	16,62537
BPZ	t7_BA2	7	168	2	BA	0,48464469	0,206965	100,0%	99,9%	0,0%	4,898	0,29461	16,62537
22BPF	t7_BA3	7	168	3	BA	0,32955751	0,149592	100,0%	100,0%	0,0%	7,12831	0,36956	19,28864
24BPF	t7_BA3	7	168	3	BA	0,67290927	0,276692	99,9%	99,9%	0,0%	7,12831	0,36956	19,28864
44BPF	t7_BA3	7	168	3	BA	0,24413114	0,396542	100,0%	99,9%	0,0%	7,12831	0,36956	19,28864
BP26DM	t7_BA3	7	168	3	BA	0,1630608	0,098169	100,0%	100,0%	0,0%	7,12831	0,36956	19,28864
BPA	t7_BA3	7	168	3	BA	8,23789196	22,34017	99,2%	96,9%	-2,2%	7,12831	0,36956	19,28864
BPAF	t7_BA3	7	168	3	BA	1,04145485	0,650619	99,9%	99,8%	-0,1%	7,12831	0,36956	19,28864
BPAP	t7_BA3	7	168	3	BA	0,32644589	0,053597	100,0%	100,0%	0,0%	7,12831	0,36956	19,28864
BPB	t7_BA3	7	168	3	BA	0,10721711	0,157594	100,0%	100,0%	0,0%	7,12831	0,36956	19,28864
BPBP	t7_BA3	7	168	3	BA	0,44016712	0,068471	100,0%	99,9%	0,0%	7,12831	0,36956	19,28864
BPC	t7_BA3	7	168	3	BA	0,4677772	0,04068	100,0%	99,9%	0,0%	7,12831	0,36956	19,28864
BPC2	t7_BA3	7	168	3	BA	0,241108	0,354406	100,0%	99,9%	0,0%	7,12831	0,36956	19,28864
BPE	t7_BA3	7	168	3	BA	0,30417752	0,131833	100,0%	100,0%	0,0%	7,12831	0,36956	19,28864
BPFL	t7_BA3	7	168	3	BA	0,60415226	1,083912	99,9%	99,8%	-0,1%	7,12831	0,36956	19,28864
BPM	t7_BA3	7	168	3	BA	0,04007608	0,090611	100,0%	100,0%	0,0%	7,12831	0,36956	19,28864
BPP	t7_BA3	7	168	3	BA	0,86762681	0,68141	99,9%	99,8%	-0,1%	7,12831	0,36956	19,28864
BPPH	t7_BA3	7	168	3	BA	0,02117467	0,06774	100,0%	100,0%	0,0%	7,12831	0,36956	19,28864
BPS	t7_BA3	7	168	3	BA	2,23485017	7,349916	99,8%	99,0%	-0,7%	7,12831	0,36956	19,28864
BPZ	t7_BA3	7	168	3	BA	0,28752238	0,11597	100,0%	100,0%	0,0%	7,12831	0,36956	19,28864
22BPF	t7_BB1	7	168	1	BB	0,40254404	0,180821	100,0%	99,9%	0,0%	0,0002	8,79729	2,27E-05
24BPF	t7_BB1	7	168	1	BB	0,94474426	0,441312	99,9%	99,9%	0,0%	0,0002	8,79729	2,27E-05
44BPF	t7_BB1	7	168	1	BB	1,66282274	0,985035	99,8%	99,7%	-0,1%	0,0002	8,79729	2,27E-05
BP26DM	t7_BB1	7	168	1	BB	0,16695891	0,069822	100,0%	100,0%	0,0%	0,0002	8,79729	2,27E-05

BPA	t7_BB1	7	168	1	BB	8,58969163	38,37296	99,1%	95,3%	-3,8%	0,0002	8,79729	2,27E-05
BPAF	t7_BB1	7	168	1	BB	2,49428225	0,24612	99,8%	99,7%	0,0%	0,0002	8,79729	2,27E-05
BPAP	t7_BB1	7	168	1	BB	0,3586031	0,035138	100,0%	100,0%	0,0%	0,0002	8,79729	2,27E-05
BPB	t7_BB1	7	168	1	BB	0,17710814	0,083779	100,0%	100,0%	0,0%	0,0002	8,79729	2,27E-05
BPBP	t7_BB1	7	168	1	BB	0,40316314	0,070951	100,0%	100,0%	0,0%	0,0002	8,79729	2,27E-05
BPC	t7_BB1	7	168	1	BB	0,04332733	0,044024	100,0%	100,0%	0,0%	0,0002	8,79729	2,27E-05
BPC2	t7_BB1	7	168	1	BB	0,49027857	0,334765	100,0%	99,9%	0,0%	0,0002	8,79729	2,27E-05
BPE	t7_BB1	7	168	1	BB	0,43157042	0,077135	100,0%	99,9%	0,0%	0,0002	8,79729	2,27E-05
BPFL	t7_BB1	7	168	1	BB	0,20755714	0,962791	100,0%	99,9%	-0,1%	0,0002	8,79729	2,27E-05
BPM	t7_BB1	7	168	1	BB	0,02488413	0,013636	100,0%	100,0%	0,0%	0,0002	8,79729	2,27E-05
BPP	t7_BB1	7	168	1	BB	0,61296873	0,764671	99,9%	99,9%	-0,1%	0,0002	8,79729	2,27E-05
BPPH	t7_BB1	7	168	1	BB	0,10648098	0,01467	100,0%	100,0%	0,0%	0,0002	8,79729	2,27E-05
BPS	t7_BB1	7	168	1	BB	8,12234884	0,40473	99,2%	99,1%	0,0%	0,0002	8,79729	2,27E-05
BPZ	t7_BB1	7	168	1	BB	0,36356248	0,060831	100,0%	100,0%	0,0%	0,0002	8,79729	2,27E-05
22BPF	t7_BB2	7	168	2	BB	0,35101788	0,503114	100,0%	99,9%	-0,1%	0,0001	9,32904	1,07E-05
24BPF	t7_BB2	7	168	2	BB	1,00412109	0,392215	99,9%	99,9%	0,0%	0,0001	9,32904	1,07E-05
44BPF	t7_BB2	7	168	2	BB	1,10416463	0,200226	99,9%	99,9%	0,0%	0,0001	9,32904	1,07E-05
BP26DM	t7_BB2	7	168	2	BB	0,12962105	0,021322	100,0%	100,0%	0,0%	0,0001	9,32904	1,07E-05
BPA	t7_BB2	7	168	2	BB	0	34,80908	100,0%	96,5%	-3,5%	0,0001	9,32904	1,07E-05
BPAF	t7_BB2	7	168	2	BB	1,13128942	0,349543	99,9%	99,9%	0,0%	0,0001	9,32904	1,07E-05
BPAP	t7_BB2	7	168	2	BB	0,3029573	0,134631	100,0%	100,0%	0,0%	0,0001	9,32904	1,07E-05
BPB	t7_BB2	7	168	2	BB	0,2211239	0,055006	100,0%	100,0%	0,0%	0,0001	9,32904	1,07E-05
BPBP	t7_BB2	7	168	2	BB	0,08335726	0,085795	100,0%	100,0%	0,0%	0,0001	9,32904	1,07E-05
BPC	t7_BB2	7	168	2	BB	0,01840193	0,047219	100,0%	100,0%	0,0%	0,0001	9,32904	1,07E-05
BPC2	t7_BB2	7	168	2	BB	0,51845219	0,208935	99,9%	99,9%	0,0%	0,0001	9,32904	1,07E-05
BPE	t7_BB2	7	168	2	BB	0,19928074	0,103786	100,0%	100,0%	0,0%	0,0001	9,32904	1,07E-05
BPFL	t7_BB2	7	168	2	BB	0,27039474	0,50858	100,0%	99,9%	-0,1%	0,0001	9,32904	1,07E-05
BPM	t7_BB2	7	168	2	BB	0,05676237	0,02652	100,0%	100,0%	0,0%	0,0001	9,32904	1,07E-05
BPP	t7_BB2	7	168	2	BB	0,85894665	0,769757	99,9%	99,8%	-0,1%	0,0001	9,32904	1,07E-05
BPPH	t7_BB2	7	168	2	BB	0,01466232	0,028047	100,0%	100,0%	0,0%	0,0001	9,32904	1,07E-05
BPS	t7_BB2	7	168	2	BB	4,7623172	2,55489	99,5%	99,3%	-0,3%	0,0001	9,32904	1,07E-05
BPZ	t7_BB2	7	168	2	BB	0,214235	0,093066	100,0%	100,0%	0,0%	0,0001	9,32904	1,07E-05
22BPF	t7_BB3	7	168	3	BB	0,20111775	0,16334	100,0%	100,0%	0,0%	0,0006	12,72094	4,72E-05
24BPF	t7_BB3	7	168	3	BB	0,92913494	0,081985	99,9%	99,9%	0,0%	0,0006	12,72094	4,72E-05
44BPF	t7_BB3	7	168	3	BB	0,10190649	0,065705	100,0%	100,0%	0,0%	0,0006	12,72094	4,72E-05
BP26DM	t7_BB3	7	168	3	BB	0,06896996	0,02	100,0%	100,0%	0,0%	0,0006	12,72094	4,72E-05
BPA	t7_BB3	7	168	3	BB	25,6525237	37,76725	97,4%	93,7%	-3,8%	0,0006	12,72094	4,72E-05
BPAF	t7_BB3	7	168	3	BB	0,53897012	0,14036	99,9%	99,9%	0,0%	0,0006	12,72094	4,72E-05
BPAP	t7_BB3	7	168	3	BB	0,06078017	0,105272	100,0%	100,0%	0,0%	0,0006	12,72094	4,72E-05
BPB	t7_BB3	7	168	3	BB	0,43646537	0,019904	100,0%	100,0%	0,0%	0,0006	12,72094	4,72E-05
BPBP	t7_BB3	7	168	3	BB	0,13654471	0,058899	100,0%	100,0%	0,0%	0,0006	12,72094	4,72E-05
BPC	t7_BB3	7	168	3	BB	0,05573465	0,02	100,0%	100,0%	0,0%	0,0006	12,72094	4,72E-05
BPC2	t7_BB3	7	168	3	BB	0,0885274	0,141169	100,0%	100,0%	0,0%	0,0006	12,72094	4,72E-05
BPE	t7_BB3	7	168	3	BB	0,40481989	0,115053	100,0%	99,9%	0,0%	0,0006	12,72094	4,72E-05
BPFL	t7_BB3	7	168	3	BB	0,4989608	0,596135	100,0%	99,9%	-0,1%	0,0006	12,72094	4,72E-05
BPM	t7_BB3	7	168	3	BB	0,04298242	0,025557	100,0%	100,0%	0,0%	0,0006	12,72094	4,72E-05
BPP	t7_BB3	7	168	3	BB	0,55801918	0,571927	99,9%	99,9%	-0,1%	0,0006	12,72094	4,72E-05
BPPH	t7_BB3	7	168	3	BB	0,03034356	0,029864	100,0%	100,0%	0,0%	0,0006	12,72094	4,72E-05
BPS	t7_BB3	7	168	3	BB	2,78046014	1,405175	99,7%	99,6%	-0,1%	0,0006	12,72094	4,72E-05
BPZ	t7_BB3	7	168	3	BB	0,12737926	0,150373	100,0%	100,0%	0,0%	0,0006	12,72094	4,72E-05
22BPF	t7_BK1	7	168	1	BK	1,35536106	0,243088	99,9%	99,8%	0,0%	9,12325	1,81417	5,028884
24BPF	t7_BK1	7	168	1	BK	1,97534034	0,196839	99,8%	99,8%	0,0%	9,12325	1,81417	5,028884
44BPF	t7_BK1	7	168	1	BK	1,74770703	0,082938	99,8%	99,8%	0,0%	9,12325	1,81417	5,028884
BP26DM	t7_BK1	7	168	1	BK	0,21435611	0,083899	100,0%	100,0%	0,0%	9,12325	1,81417	5,028884
BPA	t7_BK1	7	168	1	BK	0	24,34926	100,0%	97,6%	-2,4%	9,12325	1,81417	5,028884
BPAF	t7_BK1	7	168	1	BK	2,30352352	0,445096	99,8%	99,7%	0,0%	9,12325	1,81417	5,028884
BPAP	t7_BK1	7	168	1	BK	1,3840566	0,102866	99,9%	99,9%	0,0%	9,12325	1,81417	5,028884
BPB	t7_BK1	7	168	1	BK	1,6195418	0,215362	99,8%	99,8%	0,0%	9,12325	1,81417	5,028884
BPBP	t7_BK1	7	168	1	BK	0,97581976	0,039553	99,9%	99,9%	0,0%	9,12325	1,81417	5,028884
BPC	t7_BK1	7	168	1	BK	0,75547971	0,088218	99,9%	99,9%	0,0%	9,12325	1,81417	5,028884
BPC2	t7_BK1	7	168	1	BK	0,22558698	0,148271	100,0%	100,0%	0,0%	9,12325	1,81417	5,028884
BPE	t7_BK1	7	168	1	BK	1,37471014	0,148243	99,9%	99,8%	0,0%	9,12325	1,81417	5,028884
BPFL	t7_BK1	7	168	1	BK	2,11330975	0,460824	99,8%	99,7%	0,0%	9,12325	1,81417	5,028884
BPM	t7_BK1	7	168	1	BK	0,70203362	0,037854	99,9%	99,9%	0,0%	9,12325	1,81417	5,028884
BPP	t7_BK1	7	168	1	BK	0,9120989	0,654968	99,9%	99,8%	-0,1%	9,12325	1,81417	5,028884
BPPH	t7_BK1	7	168	1	BK	0,20063852	0,094199	100,0%	100,0%	0,0%	9,12325	1,81417	5,028884
BPS	t7_BK1	7	168	1	BK	10,2397201	1,919957	99,0%	98,8%	-0,2%	9,12325	1,81417	5,028884
BPZ	t7_BK1	7	168	1	BK	1,22255755	0,028752	99,9%	99,9%	0,0%	9,12325	1,81417	5,028884
22BPF	t7_BK2	7	168	2	BK	0,45147322	0,129126	100,0%	99,9%	0,0%	5,92565	1,95439	3,031969
24BPF	t7_BK2	7	168	2	BK	0,47200976	0,292565	100,0%	99,9%	0,0%	5,92565	1,95439	3,031969
44BPF	t7_BK2	7	168	2	BK	0,88198166	0,367595	99,9%	99,9%	0,0%	5,92565	1,95439	3,031969
BP26DM	t7_BK2	7	168	2	BK	0,03482567	0,138906	100,0%	100,0%	0,0%	5,92565	1,95439	3,031969
BPA	t7_BK2	7	168	2	BK	0	19,21848	100,0%	98,1%	-1,9%	5,92565	1,95439	3,031969
BPAF	t7_BK2	7	168	2	BK	0,68444595	0,49366	99,9%	99,9%	0,0%	5,92565	1,95439	3,031969
BPAP	t7_BK2	7	168	2	BK	0,04951029	0,318932	100,0%	100,0%	0,0%	5,92565	1,95439	3,031969
BPB	t7_BK2	7	168	2	BK	0,24731053	0,100436	100,0%	100,0%	0,0%	5,92565	1,95439	3,031969
BPBP	t7_BK2	7	168	2	BK	0,16061308	0,07827	100,0%	100,0%	0,0%	5,92565	1,95439	3,031969
BPC	t7_BK2	7	168	2	BK	0,08093668	0,05	100,0%	100,0%	0,0%	5,92565	1,95439	3,031969
BPC2	t7_BK2	7	168	2	BK	0,35679004	0,063218	100,0%	100,0%	0,0%	5,92565	1,95439	3,031969
BPE	t7_BK2	7	168	2	BK	0,32567412	0,01634	100,0%	100,0%	0,0%	5,92565	1,95439	3,031969
BPFL	t7_BK2	7	168	2	BK	0,27492873	0,130912	100,0%	100,0%	0,0%	5,92565	1,95439	3,031969
BPM	t7_BK2	7	168	2	BK	0,06313254	0,015822	100,0%	100,0%	0,0%	5,92565	1,95439	3,031969
BPP	t7_BK2	7	168	2	BK	0,47991934	0,763515	100,0%	99,9%	-0,1%	5,92565	1,95439	3,031969
BPPH	t7_BK2	7	168	2	BK	0,05160613	0,064748	100,0%	100,0%	0,0%	5,92565	1,95439	3,031969
BPS	t7_BK2	7	168	2	BK	4,64775956	1,026669	99,5%	99,4%	-0,1%	5,92565	1,95439	3,031969
BPZ	t7_BK2	7	168	2	BK	0,07132019	0,097738	100,0%	100,0%	0,0%	5,92565	1,95439	3,031969

22BPF	t7_BK3	7	168	3	BK	0,168051	0,334284	100,0%	99,9%	0,0%	6,80818	2,10669	3,231695
24BPF	t7_BK3	7	168	3	BK	0,31363447	0,349138	100,0%	99,9%	0,0%	6,80818	2,10669	3,231695
44BPF	t7_BK3	7	168	3	BK	0,15	0,24543	100,0%	100,0%	0,0%	6,80818	2,10669	3,231695
BP26DM	t7_BK3	7	168	3	BK	0,04398837	0,042245	100,0%	100,0%	0,0%	6,80818	2,10669	3,231695
BPA	t7_BK3	7	168	3	BK	0	25,22445	100,0%	97,5%	-2,5%	6,80818	2,10669	3,231695
BPAF	t7_BK3	7	168	3	BK	0,74570551	0,121708	99,9%	99,9%	0,0%	6,80818	2,10669	3,231695
BPAP	t7_BK3	7	168	3	BK	0,29126634	0,082319	100,0%	100,0%	0,0%	6,80818	2,10669	3,231695
BPB	t7_BK3	7	168	3	BK	0,16111069	0,279325	100,0%	100,0%	0,0%	6,80818	2,10669	3,231695
BPBP	t7_BK3	7	168	3	BK	0,09167391	0,024407	100,0%	100,0%	0,0%	6,80818	2,10669	3,231695
BPC	t7_BK3	7	168	3	BK	0,03821308	0,05	100,0%	100,0%	0,0%	6,80818	2,10669	3,231695
BPC2	t7_BK3	7	168	3	BK	0,95603288	0,506256	99,9%	99,9%	-0,1%	6,80818	2,10669	3,231695
BPE	t7_BK3	7	168	3	BK	0,347546	0,092792	100,0%	100,0%	0,0%	6,80818	2,10669	3,231695
BPFL	t7_BK3	7	168	3	BK	0,09238402	0,846602	100,0%	99,9%	-0,1%	6,80818	2,10669	3,231695
BPM	t7_BK3	7	168	3	BK	0,02346893	0,027696	100,0%	100,0%	0,0%	6,80818	2,10669	3,231695
BPP	t7_BK3	7	168	3	BK	0,65356543	0,637355	99,9%	99,9%	-0,1%	6,80818	2,10669	3,231695
BPPH	t7_BK3	7	168	3	BK	0,1322221	0,056819	100,0%	100,0%	0,0%	6,80818	2,10669	3,231695
BPS	t7_BK3	7	168	3	BK	2,03042613	2,5197	99,8%	99,5%	-0,3%	6,80818	2,10669	3,231695
BPZ	t7_BK3	7	168	3	BK	0,13595282	0,081049	100,0%	100,0%	0,0%	6,80818	2,10669	3,231695
22BPF	t7_EA1	7	168	1	EA	771,217089	7,930571	22,9%	22,1%	-0,8%	8,4981	0,87086	9,758285
24BPF	t7_EA1	7	168	1	EA	722,050217	23,23052	27,8%	25,5%	-2,3%	8,4981	0,87086	9,758285
44BPF	t7_EA1	7	168	1	EA	719,185016	14,21484	28,1%	26,7%	-1,4%	8,4981	0,87086	9,758285
BP26DM	t7_EA1	7	168	1	EA	466,968931	21,59282	53,3%	51,1%	-2,2%	8,4981	0,87086	9,758285
BPA	t7_EA1	7	168	1	EA	671,210644	43,45557	32,9%	28,5%	-4,3%	8,4981	0,87086	9,758285
BPAF	t7_EA1	7	168	1	EA	793,129503	13,88137	20,7%	19,3%	-1,4%	8,4981	0,87086	9,758285
BPAP	t7_EA1	7	168	1	EA	680,121957	31,90132	32,0%	28,8%	-3,2%	8,4981	0,87086	9,758285
BPB	t7_EA1	7	168	1	EA	712,128272	24,59668	28,8%	26,3%	-2,5%	8,4981	0,87086	9,758285
BPBP	t7_EA1	7	168	1	EA	360,998361	178,6322	63,9%	46,0%	-17,9%	8,4981	0,87086	9,758285
BPC	t7_EA1	7	168	1	EA	633,408981	34,96005	36,7%	33,2%	-3,5%	8,4981	0,87086	9,758285
BPC2	t7_EA1	7	168	1	EA	23,9379893	1,151169	97,6%	97,5%	-0,1%	8,4981	0,87086	9,758285
BPE	t7_EA1	7	168	1	EA	717,041149	22,49853	28,3%	26,0%	-2,2%	8,4981	0,87086	9,758285
BPFL	t7_EA1	7	168	1	EA	328,059194	151,5131	67,2%	52,0%	-15,2%	8,4981	0,87086	9,758285
BPM	t7_EA1	7	168	1	EA	271,848074	164,3945	72,8%	56,4%	-16,4%	8,4981	0,87086	9,758285
BPP	t7_EA1	7	168	1	EA	149,09343	234,6088	85,1%	61,6%	-23,5%	8,4981	0,87086	9,758285
BPPH	t7_EA1	7	168	1	EA	26,2145996	168,4154	97,4%	80,5%	-16,8%	8,4981	0,87086	9,758285
BPS	t7_EA1	7	168	1	EA	661,374232	17,45974	33,9%	32,1%	-1,7%	8,4981	0,87086	9,758285
BPZ	t7_EA1	7	168	1	EA	658,218257	37,78149	34,2%	30,4%	-3,8%	8,4981	0,87086	9,758285
22BPF	t7_EA2	7	168	2	EA	766,833178	6,256201	23,3%	22,7%	-0,6%	3,59481	0,51537	6,975202
24BPF	t7_EA2	7	168	2	EA	726,175552	13,93583	27,4%	26,0%	-1,4%	3,59481	0,51537	6,975202
44BPF	t7_EA2	7	168	2	EA	724,758768	8,99748	27,5%	26,6%	-0,9%	3,59481	0,51537	6,975202
BP26DM	t7_EA2	7	168	2	EA	362,699972	14,78862	63,7%	62,3%	-1,5%	3,59481	0,51537	6,975202
BPA	t7_EA2	7	168	2	EA	737,25341	35,02851	26,3%	22,8%	-3,5%	3,59481	0,51537	6,975202
BPAF	t7_EA2	7	168	2	EA	775,234571	9,171807	22,5%	21,6%	-0,9%	3,59481	0,51537	6,975202
BPAP	t7_EA2	7	168	2	EA	681,315862	18,93406	31,9%	30,0%	-1,9%	3,59481	0,51537	6,975202
BPB	t7_EA2	7	168	2	EA	755,869803	16,77142	24,4%	22,7%	-1,7%	3,59481	0,51537	6,975202
BPBP	t7_EA2	7	168	2	EA	415,510591	93,89761	58,4%	49,1%	-9,4%	3,59481	0,51537	6,975202
BPC	t7_EA2	7	168	2	EA	626,832739	23,5104	37,3%	35,0%	-2,4%	3,59481	0,51537	6,975202
BPC2	t7_EA2	7	168	2	EA	1,16314917	0,107828	99,9%	99,9%	0,0%	3,59481	0,51537	6,975202
BPE	t7_EA2	7	168	2	EA	746,110227	12,87602	25,4%	24,1%	-1,3%	3,59481	0,51537	6,975202
BPFL	t7_EA2	7	168	2	EA	402,549382	88,08643	59,7%	50,9%	-8,8%	3,59481	0,51537	6,975202
BPM	t7_EA2	7	168	2	EA	318,442557	118,5822	68,2%	56,3%	-11,9%	3,59481	0,51537	6,975202
BPP	t7_EA2	7	168	2	EA	195,873586	155,285	80,4%	64,9%	-15,5%	3,59481	0,51537	6,975202
BPPH	t7_EA2	7	168	2	EA	29,72996	89,41293	97,0%	88,1%	-8,9%	3,59481	0,51537	6,975202
BPS	t7_EA2	7	168	2	EA	665,210316	19,0683	33,5%	31,6%	-1,9%	3,59481	0,51537	6,975202
BPZ	t7_EA2	7	168	2	EA	706,00897	25,81209	29,4%	26,8%	-2,6%	3,59481	0,51537	6,975202
22BPF	t7_EA3	7	168	3	EA	877,268766	5,618305	12,3%	11,7%	-0,6%	3,68822	0,61287	6,017948
24BPF	t7_EA3	7	168	3	EA	849,202939	13,80635	15,1%	13,7%	-1,4%	3,68822	0,61287	6,017948
44BPF	t7_EA3	7	168	3	EA	774,657121	9,737896	22,5%	21,6%	-1,0%	3,68822	0,61287	6,017948
BP26DM	t7_EA3	7	168	3	EA	384,560229	15,97239	61,5%	59,9%	-1,6%	3,68822	0,61287	6,017948
BPA	t7_EA3	7	168	3	EA	787,115093	33,68615	21,3%	17,9%	-3,4%	3,68822	0,61287	6,017948
BPAF	t7_EA3	7	168	3	EA	869,791456	9,153251	13,0%	12,1%	-0,9%	3,68822	0,61287	6,017948
BPAP	t7_EA3	7	168	3	EA	763,112243	19,51306	23,7%	21,7%	-2,0%	3,68822	0,61287	6,017948
BPB	t7_EA3	7	168	3	EA	778,274486	18,66037	22,2%	20,3%	-1,9%	3,68822	0,61287	6,017948
BPBP	t7_EA3	7	168	3	EA	474,038682	86,66049	52,6%	43,9%	-8,7%	3,68822	0,61287	6,017948
BPC	t7_EA3	7	168	3	EA	667,073522	25,20342	33,3%	30,8%	-2,5%	3,68822	0,61287	6,017948
BPC2	t7_EA3	7	168	3	EA	0,81524872	0,262339	99,9%	99,9%	0,0%	3,68822	0,61287	6,017948
BPE	t7_EA3	7	168	3	EA	845,60226	13,32678	15,4%	14,1%	-1,3%	3,68822	0,61287	6,017948
BPFL	t7_EA3	7	168	3	EA	438,651089	70,54224	56,1%	49,1%	-7,1%	3,68822	0,61287	6,017948
BPM	t7_EA3	7	168	3	EA	338,883464	111,2335	66,1%	55,0%	-11,1%	3,68822	0,61287	6,017948
BPP	t7_EA3	7	168	3	EA	230,347262	153,5284	77,0%	61,6%	-15,4%	3,68822	0,61287	6,017948
BPPH	t7_EA3	7	168	3	EA	40,712022	104,2028	95,9%	85,5%	-10,4%	3,68822	0,61287	6,017948
BPS	t7_EA3	7	168	3	EA	673,911841	13,24038	32,6%	31,3%	-1,3%	3,68822	0,61287	6,017948
BPZ	t7_EA3	7	168	3	EA	723,707924	26,08097	27,6%	25,0%	-2,6%	3,68822	0,61287	6,017948
22BPF	t7_EB1	7	168	1	EB	802,432644	14,88552	19,8%	18,3%	-1,5%	0,0014	7,50599	0,000187
24BPF	t7_EB1	7	168	1	EB	767,404365	10,13126	23,3%	22,2%	-1,0%	0,0014	7,50599	0,000187
44BPF	t7_EB1	7	168	1	EB	782,256513	10,2783	21,8%	20,7%	-1,0%	0,0014	7,50599	0,000187
BP26DM	t7_EB1	7	168	1	EB	736,479411	25,69034	26,4%	23,8%	-2,6%	0,0014	7,50599	0,000187
BPA	t7_EB1	7	168	1	EB	556,857958	31,94586	44,3%	41,1%	-3,2%	0,0014	7,50599	0,000187
BPAF	t7_EB1	7	168	1	EB	721,050799	58,34529	27,9%	22,1%	-5,8%	0,0014	7,50599	0,000187
BPAP	t7_EB1	7	168	1	EB	696,504799	38,8617	30,3%	26,5%	-3,9%	0,0014	7,50599	0,000187
BPB	t7_EB1	7	168	1	EB	718,536549	12,15333	28,1%	26,9%	-1,2%	0,0014	7,50599	0,000187
BPBP	t7_EB1	7	168	1	EB	304,872642	220,1874	69,5%	47,5%	-22,0%	0,0014	7,50599	0,000187
BPC	t7_EB1	7	168	1	EB	720,312748	17,66233	28,0%	26,2%	-1,8%	0,0014	7,50599	0,000187
BPC2	t7_EB1	7	168	1	EB	599,934626	42,97521	40,0%	35,7%	-4,3%	0,0014	7,50599	0,000187
BPE	t7_EB1	7	168	1	EB	681,342698	7,818194	31,9%	31,1%	-0,8%	0,0014	7,50599	0,000187
BPFL	t7_EB1	7	168	1	EB	326,585206	175,2873	67,3%	49,8%	-17,5%	0,0014	7,50599	0,000187
BPM	t7_EB1	7	168	1	EB	149,196716	166,7465	85,1%	68,4%	-16,7%	0,0014	7,50599	0,000187

BPP	t7_EB1	7	168	1	EB	76,9972768	187,2086	92,3%	73,6%	-18,7%	0,0014	7,50599	0,000187
BPPH	t7_EB1	7	168	1	EB	58,6918833	332,055	94,1%	60,9%	-33,2%	0,0014	7,50599	0,000187
BPS	t7_EB1	7	168	1	EB	770,026168	7,803304	23,0%	22,2%	-0,8%	0,0014	7,50599	0,000187
BPZ	t7_EB1	7	168	1	EB	732,782949	35,94343	26,7%	23,1%	-3,6%	0,0014	7,50599	0,000187
22BPF	t7_EB2	7	168	2	EB	759,970183	12,59011	24,0%	22,7%	-1,3%	0,0005	7,81457	6,4E-05
24BPF	t7_EB2	7	168	2	EB	736,715689	9,916299	26,3%	25,3%	-1,0%	0,0005	7,81457	6,4E-05
44BPF	t7_EB2	7	168	2	EB	730,624787	7,678146	26,9%	26,2%	-0,8%	0,0005	7,81457	6,4E-05
BP26DM	t7_EB2	7	168	2	EB	703,094961	27,08967	29,7%	27,0%	-2,7%	0,0005	7,81457	6,4E-05
BPA	t7_EB2	7	168	2	EB	534,210021	25,26449	46,6%	44,1%	-2,5%	0,0005	7,81457	6,4E-05
BPAF	t7_EB2	7	168	2	EB	669,970023	58,18041	33,0%	27,2%	-5,8%	0,0005	7,81457	6,4E-05
BPAP	t7_EB2	7	168	2	EB	658,514902	37,47838	34,1%	30,4%	-3,7%	0,0005	7,81457	6,4E-05
BPB	t7_EB2	7	168	2	EB	678,913909	11,43371	32,1%	31,0%	-1,1%	0,0005	7,81457	6,4E-05
BPBP	t7_EB2	7	168	2	EB	291,387848	218,4751	70,9%	49,0%	-21,8%	0,0005	7,81457	6,4E-05
BPC	t7_EB2	7	168	2	EB	670,138659	18,65304	33,0%	31,1%	-1,9%	0,0005	7,81457	6,4E-05
BPC2	t7_EB2	7	168	2	EB	533,083358	41,83234	46,7%	42,5%	-4,2%	0,0005	7,81457	6,4E-05
BPE	t7_EB2	7	168	2	EB	642,517447	7,18019	35,7%	35,0%	-0,7%	0,0005	7,81457	6,4E-05
BPFL	t7_EB2	7	168	2	EB	299,500312	156,4261	70,0%	54,4%	-15,6%	0,0005	7,81457	6,4E-05
BPM	t7_EB2	7	168	2	EB	138,786193	161,3028	86,1%	70,0%	-16,1%	0,0005	7,81457	6,4E-05
BPP	t7_EB2	7	168	2	EB	72,117615	175,4213	92,8%	75,2%	-17,5%	0,0005	7,81457	6,4E-05
BPPH	t7_EB2	7	168	2	EB	54,2071972	319,5082	94,6%	62,6%	-32,0%	0,0005	7,81457	6,4E-05
BPS	t7_EB2	7	168	2	EB	758,915343	4,183076	24,1%	23,7%	-0,4%	0,0005	7,81457	6,4E-05
BPZ	t7_EB2	7	168	2	EB	712,217493	35,13937	28,8%	25,3%	-3,5%	0,0005	7,81457	6,4E-05
22BPF	t7_EB3	7	168	3	EB	711,833017	11,1336	28,8%	27,7%	-1,1%	0,0006	8,58932	6,99E-05
24BPF	t7_EB3	7	168	3	EB	695,806696	6,679247	30,4%	29,8%	-0,7%	0,0006	8,58932	6,99E-05
44BPF	t7_EB3	7	168	3	EB	701,019526	5,214523	29,9%	29,4%	-0,5%	0,0006	8,58932	6,99E-05
BP26DM	t7_EB3	7	168	3	EB	707,493959	24,43557	29,3%	26,8%	-2,4%	0,0006	8,58932	6,99E-05
BPA	t7_EB3	7	168	3	EB	490,289186	32,6281	51,0%	47,7%	-3,3%	0,0006	8,58932	6,99E-05
BPAF	t7_EB3	7	168	3	EB	640,852486	59,89391	35,9%	29,9%	-6,0%	0,0006	8,58932	6,99E-05
BPAP	t7_EB3	7	168	3	EB	639,101475	37,17198	36,1%	32,4%	-3,7%	0,0006	8,58932	6,99E-05
BPB	t7_EB3	7	168	3	EB	643,357606	10,97521	35,7%	34,6%	-1,1%	0,0006	8,58932	6,99E-05
BPBP	t7_EB3	7	168	3	EB	268,53119	207,293	73,1%	52,4%	-20,7%	0,0006	8,58932	6,99E-05
BPC	t7_EB3	7	168	3	EB	655,536332	15,60574	34,4%	32,9%	-1,6%	0,0006	8,58932	6,99E-05
BPC2	t7_EB3	7	168	3	EB	536,937861	37,79289	46,3%	42,5%	-3,8%	0,0006	8,58932	6,99E-05
BPE	t7_EB3	7	168	3	EB	591,411268	5,043628	40,9%	40,4%	-0,5%	0,0006	8,58932	6,99E-05
BPFL	t7_EB3	7	168	3	EB	282,226844	156,1636	71,8%	56,2%	-15,6%	0,0006	8,58932	6,99E-05
BPM	t7_EB3	7	168	3	EB	122,652262	140,5869	87,7%	73,7%	-14,1%	0,0006	8,58932	6,99E-05
BPP	t7_EB3	7	168	3	EB	62,5770292	155,0164	93,7%	78,2%	-15,5%	0,0006	8,58932	6,99E-05
BPPH	t7_EB3	7	168	3	EB	54,8353695	264,344	94,5%	68,1%	-26,4%	0,0006	8,58932	6,99E-05
BPS	t7_EB3	7	168	3	EB	756,062814	2,866932	24,4%	24,1%	-0,3%	0,0006	8,58932	6,99E-05
BPZ	t7_EB3	7	168	3	EB	688,514627	33,77546	31,1%	27,8%	-3,4%	0,0006	8,58932	6,99E-05
22BPF	t7_EK1	7	168	1	EK	748,294208	8,670182	25,2%	24,3%	-0,9%	9,12116	1,72675	5,28227
24BPF	t7_EK1	7	168	1	EK	635,618998	27,45921	36,4%	33,7%	-2,7%	9,12116	1,72675	5,28227
44BPF	t7_EK1	7	168	1	EK	629,65517	15,18407	37,0%	35,5%	-1,5%	9,12116	1,72675	5,28227
BP26DM	t7_EK1	7	168	1	EK	170,095955	22,45857	83,0%	80,7%	-2,2%	9,12116	1,72675	5,28227
BPA	t7_EK1	7	168	1	EK	622,891526	60,77271	37,7%	31,6%	-6,1%	9,12116	1,72675	5,28227
BPAF	t7_EK1	7	168	1	EK	793,07574	17,32324	20,7%	19,0%	-1,7%	9,12116	1,72675	5,28227
BPAP	t7_EK1	7	168	1	EK	654,679885	55,52876	34,5%	29,0%	-5,6%	9,12116	1,72675	5,28227
BPB	t7_EK1	7	168	1	EK	683,438655	41,71048	31,7%	27,5%	-4,2%	9,12116	1,72675	5,28227
BPBP	t7_EK1	7	168	1	EK	317,934757	209,4861	68,2%	47,3%	-20,9%	9,12116	1,72675	5,28227
BPC	t7_EK1	7	168	1	EK	451,188632	50,98979	54,9%	49,8%	-5,1%	9,12116	1,72675	5,28227
BPC2	t7_EK1	7	168	1	EK	0,29347915	0,265447	100,0%	99,9%	0,0%	9,12116	1,72675	5,28227
BPE	t7_EK1	7	168	1	EK	666,507126	23,71317	33,3%	31,0%	-2,4%	9,12116	1,72675	5,28227
BPFL	t7_EK1	7	168	1	EK	303,766708	186,4579	69,6%	51,0%	-18,6%	9,12116	1,72675	5,28227
BPM	t7_EK1	7	168	1	EK	208,482993	179,5361	79,2%	61,2%	-18,0%	9,12116	1,72675	5,28227
BPP	t7_EK1	7	168	1	EK	116,452288	204,7872	88,4%	67,9%	-20,5%	9,12116	1,72675	5,28227
BPPH	t7_EK1	7	168	1	EK	29,7847947	201,0009	97,0%	76,9%	-20,1%	9,12116	1,72675	5,28227
BPS	t7_EK1	7	168	1	EK	584,357732	9,86919	41,6%	40,6%	-1,0%	9,12116	1,72675	5,28227
BPZ	t7_EK1	7	168	1	EK	594,896384	65,89899	40,5%	33,9%	-6,6%	9,12116	1,72675	5,28227
22BPF	t7_EK2	7	168	2	EK	685,428305	7,254831	31,5%	30,7%	-0,7%	6,37355	2,11816	3,009003
24BPF	t7_EK2	7	168	2	EK	566,57541	19,36754	43,3%	41,4%	-1,9%	6,37355	2,11816	3,009003
44BPF	t7_EK2	7	168	2	EK	600,316889	12,78437	40,0%	38,7%	-1,3%	6,37355	2,11816	3,009003
BP26DM	t7_EK2	7	168	2	EK	47,5083156	8,275856	95,2%	94,4%	-0,8%	6,37355	2,11816	3,009003
BPA	t7_EK2	7	168	2	EK	623,078853	47,704	37,7%	32,9%	-4,8%	6,37355	2,11816	3,009003
BPAF	t7_EK2	7	168	2	EK	768,994068	18,88698	23,1%	21,2%	-1,9%	6,37355	2,11816	3,009003
BPAP	t7_EK2	7	168	2	EK	653,767968	64,67823	34,6%	28,2%	-6,5%	6,37355	2,11816	3,009003
BPB	t7_EK2	7	168	2	EK	642,878939	40,26198	35,7%	31,7%	-4,0%	6,37355	2,11816	3,009003
BPBP	t7_EK2	7	168	2	EK	343,318871	208,8014	65,7%	44,8%	-20,9%	6,37355	2,11816	3,009003
BPC	t7_EK2	7	168	2	EK	333,619175	35,74295	66,6%	63,1%	-3,6%	6,37355	2,11816	3,009003
BPC2	t7_EK2	7	168	2	EK	1,2162865	0,297803	99,9%	99,8%	0,0%	6,37355	2,11816	3,009003
BPE	t7_EK2	7	168	2	EK	619,041731	18,88009	38,1%	36,2%	-1,9%	6,37355	2,11816	3,009003
BPFL	t7_EK2	7	168	2	EK	367,319696	225,8201	63,3%	40,7%	-22,6%	6,37355	2,11816	3,009003
BPM	t7_EK2	7	168	2	EK	240,396327	196,9706	76,0%	56,3%	-19,7%	6,37355	2,11816	3,009003
BPP	t7_EK2	7	168	2	EK	139,679721	220,2768	86,0%	64,0%	-22,0%	6,37355	2,11816	3,009003
BPPH	t7_EK2	7	168	2	EK	35,7973201	197,9338	96,4%	76,6%	-19,8%	6,37355	2,11816	3,009003
BPS	t7_EK2	7	168	2	EK	657,477878	5,594875	34,3%	33,7%	-0,6%	6,37355	2,11816	3,009003
BPZ	t7_EK2	7	168	2	EK	647,190866	67,07116	35,3%	28,6%	-6,7%	6,37355	2,11816	3,009003
22BPF	t7_EK3	7	168	3	EK	733,271888	10,22277	26,7%	25,7%	-1,0%	7,35725	1,9023	3,867555
24BPF	t7_EK3	7	168	3	EK	585,556156	23,44774	41,4%	39,1%	-2,3%	7,35725	1,9023	3,867555
44BPF	t7_EK3	7	168	3	EK	616,061475	15,37562	38,4%	36,9%	-1,5%	7,35725	1,9023	3,867555
BP26DM	t7_EK3	7	168	3	EK	69,2702206	9,422751	93,1%	92,1%	-0,9%	7,35725	1,9023	3,867555
BPA	t7_EK3	7	168	3	EK	641,664297	93,75296	35,8%	26,5%	-9,4%	7,35725	1,9023	3,867555
BPAF	t7_EK3	7	168	3	EK	809,861506	22,18927	19,0%	16,8%	-2,2%	7,35725	1,9023	3,867555
BPAP	t7_EK3	7	168	3	EK	659,799565	65,08708	34,0%	27,5%	-6,5%	7,35725	1,9023	3,867555
BPB	t7_EK3	7	168	3	EK	653,495334	40,94774	34,7%	30,6%	-4,1%	7,35725	1,9023	3,867555
BPBP	t7_EK3	7	168	3	EK	301,268384	201,9527	69,9%	49,7%	-20,2%	7,35725	1,9023	3,867555
BPC	t7_EK3	7	168	3	EK	361,689795	39,38958	63,8%	59,9%	-3,9%	7,35725	1,9023	3,867555

BPC2	t7_EK3	7	168	3	EK	0,74078582	0,282622	99,9%	99,9%	0,0%	7,35725	1,9023	3,867555
BPE	t7_EK3	7	168	3	EK	642,423324	24,09817	35,8%	33,3%	-2,4%	7,35725	1,9023	3,867555
BPFL	t7_EK3	7	168	3	EK	314,257516	214,3244	68,6%	47,1%	-21,4%	7,35725	1,9023	3,867555
BPM	t7_EK3	7	168	3	EK	216,646519	183,8652	78,3%	59,9%	-18,4%	7,35725	1,9023	3,867555
BPP	t7_EK3	7	168	3	EK	120,669197	204,9103	87,9%	67,4%	-20,5%	7,35725	1,9023	3,867555
BPPH	t7_EK3	7	168	3	EK	28,7334446	175,2602	97,1%	79,6%	-17,5%	7,35725	1,9023	3,867555
BPS	t7_EK3	7	168	3	EK	653,818497	11,26483	34,6%	33,5%	-1,1%	7,35725	1,9023	3,867555
BPZ	t7_EK3	7	168	3	EK	585,483866	67,78093	41,5%	34,7%	-6,8%	7,35725	1,9023	3,867555

Table S3. GenBank (NCBI) output for identification of Gram-negative bacilli isolated from microalgal cultures. Query coverage, E value and percent identity for the 16S ribosomal RNA contig of both bacterial isolates.

Description	Scientific Name	Max Score	Total Score	Query Cover	E value	Per. Ident	Acc. Len	Accession
Thalassospira sp. AU193_Ted1_R2A partial 16S rRNA gene, isolate AU193_Ted1_R2A	Thalassospira sp. AU193_Ted1_R2A	695	695	99%	0.0	99.47%	735	LN909101.1
Thalassospira xiamenensis strain CSE_33 16S ribosomal RNA gene, partial sequence	Thalassospira xiamenensis	695	695	99%	0.0	99.47%	1386	KX027359.1
Thalassospira permensis strain CSE_28 16S ribosomal RNA gene, partial sequence	Thalassospira permensis	695	695	99%	0.0	99.47%	1400	KX027356.1
Thalassospira permensis strain CSE_27 16S ribosomal RNA gene, partial sequence	Thalassospira permensis	695	695	99%	0.0	99.47%	1382	KX027355.1
Thalassospira sp. CSE_4 16S ribosomal RNA gene, partial sequence	Thalassospira sp. CSE_4	695	695	99%	0.0	99.47%	1391	KX027340.1
Thalassospira sp. CPA-6 16S ribosomal RNA gene, partial sequence	Thalassospira sp. CPA-6	695	695	99%	0.0	99.47%	970	KT324754.1
Thalassospira sp. CPA-4 16S ribosomal RNA gene, partial sequence	Thalassospira sp. CPA-4	695	695	99%	0.0	99.47%	999	KT324752.1
Thalassospira sp. C2 16S ribosomal RNA gene, partial sequence	Thalassospira sp. C2	695	695	99%	0.0	99.47%	750	KT715766.1
Thalassospira sp. R18-4 16S ribosomal RNA gene, partial sequence	Thalassospira sp. R18-4	695	695	99%	0.0	99.47%	1384	KT429721.1
Uncultured bacterium partial 16S rRNA gene, clone EE3-1_F09	uncultured bacterium	695	695	99%	0.0	99.47%	955	LM651980.1
Thalassospira permensis strain I-A-E-11 16S ribosomal RNA gene, partial sequence	Thalassospira permensis	695	695	99%	0.0	99.47%	1397	KT922024.1
Thalassospira sp. H2-74 16S ribosomal RNA gene, partial sequence	Thalassospira sp. H2-74	695	695	99%	0.0	99.47%	1276	KM979195.1
Thalassospira sp. R-52913 16S ribosomal RNA gene, partial sequence	Thalassospira sp. R-52913	695	695	99%	0.0	99.47%	1465	KT185151.1
Thalassospira sp. R-52699 16S ribosomal RNA gene, partial sequence	Thalassospira sp. R-52699	695	695	99%	0.0	99.47%	1408	KT185150.1
Thalassospira sp. AMJ01 16S ribosomal RNA gene, partial sequence	Thalassospira sp. AMJ01	695	695	99%	0.0	99.47%	1350	KT443869.1
Thalassospira sp. SLG510B2-7-1 16S ribosomal RNA gene, partial sequence	Thalassospira sp. SLG510B2-7-1	695	695	99%	0.0	99.47%	1138	KP711622.1
Uncultured marine bacterium clone BS_CoastalExp_T55h_A_F2 16S ribosomal RNA gene, partial sequence	uncultured marine bacterium	695	695	99%	0.0	99.47%	1127	KR054235.1
Uncultured deep-sea bacterium clone Lau26_TahiMoana 16S ribosomal RNA gene, partial sequence	uncultured deep-sea bacterium	695	695	99%	0.0	99.47%	900	KP005030.1
Marine bacterium VA003 16S ribosomal RNA gene, partial sequence	marine bacterium VA003	695	695	99%	0.0	99.47%	1338	KJ814519.1
Bacterium RFB C06 partial 16S rRNA gene, isolate RFB C06	bacterium RFB C06	695	695	99%	0.0	99.47%	1229	LN681278.1
Thalassospira xiamenensis M-5 = DSM 17429, complete genome	Thalassospira xiamenensis M-5 = DSM 17429	695	2781	99%	0.0	99.47%	4576570	CP004388.1
Thalassospira permensis strain ROD131 16S ribosomal RNA gene, partial sequence	Thalassospira permensis	695	695	99%	0.0	99.47%	1369	ON878087.1
Thalassospira sp. strain B31-4 16S ribosomal RNA gene, partial sequence	Thalassospira sp.	695	695	99%	0.0	99.47%	1289	ON619592.1

Description	Scientific Name	Max Score	Total Score	Query Cover	E value	Per. Ident	Acc. Len	Accession
Thalassospira sp. strain B30-1 16S ribosomal RNA gene, partial sequence	Thalassospira sp.	695	695	99%	0.0	99.47%	1490	ON600594.1
Thalassospiraceae bacterium SW-3-3 chromosome, complete genome	Thalassospiraceae bacterium SW-3-3	695	2781	99%	0.0	99.47%	4764478	CP091912.1
Thalassospira sp. MCCC1A07747 16S ribosomal RNA gene, partial sequence	Thalassospira sp. MCCC1A07747	695	695	99%	0.0	99.47%	924	KJ956978.1
Thalassospira sp. MCCC1A07528 16S ribosomal RNA gene, partial sequence	Thalassospira sp. MCCC1A07528	695	695	99%	0.0	99.47%	930	KJ956945.1
Thalassospira sp. MCCC1A07749 16S ribosomal RNA gene, partial sequence	Thalassospira sp. MCCC1A07749	695	695	99%	0.0	99.47%	929	KJ956937.1
Thalassospira permensis genomic DNA containing 16S-23S intergenic spacer region, strain R9	Thalassospira permensis	695	695	99%	0.0	99.47%	991	LK934702.1
Uncultured bacterium clone HXF_1_83 16S ribosomal RNA gene, partial sequence	uncultured bacterium	695	695	99%	0.0	99.47%	1457	KJ814181.1
Uncultured bacterium clone HXF_1_81 16S ribosomal RNA gene, partial sequence	uncultured bacterium	695	695	99%	0.0	99.47%	1457	KJ814179.1
Uncultured bacterium clone HXF_1_71 16S ribosomal RNA gene, partial sequence	uncultured bacterium	695	695	99%	0.0	99.47%	1457	KJ814171.1
Uncultured bacterium clone HXF_1_70 16S ribosomal RNA gene, partial sequence	uncultured bacterium	695	695	99%	0.0	99.47%	1456	KJ814170.1
Uncultured bacterium clone HXF_1_67 16S ribosomal RNA gene, partial sequence	uncultured bacterium	695	695	99%	0.0	99.47%	1456	KJ814168.1
Uncultured bacterium clone HXF_1_55 16S ribosomal RNA gene, partial sequence	uncultured bacterium	695	695	99%	0.0	99.47%	1456	KJ814159.1
Uncultured bacterium clone HXF_1_48 16S ribosomal RNA gene, partial sequence	uncultured bacterium	695	695	99%	0.0	99.47%	1456	KJ814153.1
Uncultured bacterium clone HXF_1_37 16S ribosomal RNA gene, partial sequence	uncultured bacterium	695	695	99%	0.0	99.47%	1456	KJ814144.1
Uncultured bacterium clone HXF_1_35 16S ribosomal RNA gene, partial sequence	uncultured bacterium	695	695	99%	0.0	99.47%	1458	KJ814142.1
Uncultured bacterium clone HXF_1_17 16S ribosomal RNA gene, partial sequence	uncultured bacterium	695	695	99%	0.0	99.47%	1457	KJ814127.1
Uncultured bacterium clone HXF_1_105 16S ribosomal RNA gene, partial sequence	uncultured bacterium	695	695	99%	0.0	99.47%	1457	KJ814117.1
Uncultured bacterium clone HXF_1_101 16S ribosomal RNA gene, partial sequence	uncultured bacterium	695	695	99%	0.0	99.47%	1456	KJ814114.1
Uncultured bacterium clone HXF_2_85 16S ribosomal RNA gene, partial sequence	uncultured bacterium	695	695	99%	0.0	99.47%	1432	KJ814099.1
Uncultured bacterium clone HXF_2_71 16S ribosomal RNA gene, partial sequence	uncultured bacterium	695	695	99%	0.0	99.47%	1456	KJ814090.1
Uncultured bacterium clone HXF_2_42 16S ribosomal RNA gene, partial sequence	uncultured bacterium	695	695	99%	0.0	99.47%	1457	KJ814063.1
Uncultured bacterium clone HXF_2_33 16S ribosomal RNA gene, partial sequence	uncultured bacterium	695	695	99%	0.0	99.47%	1457	KJ814055.1
Uncultured bacterium clone HXF_2_30 16S ribosomal RNA gene, partial sequence	uncultured bacterium	695	695	99%	0.0	99.47%	1457	KJ814053.1
Uncultured bacterium clone HXF_2_16 16S ribosomal RNA gene, partial sequence	uncultured bacterium	695	695	99%	0.0	99.47%	1456	KJ814037.1
